# Experiences of informal caregivers supporting individuals with upper gastrointestinal cancers: a systematic review

**DOI:** 10.1186/s12913-024-11306-3

**Published:** 2024-08-14

**Authors:** Melinda Furtado, Dawn Davis, Jenny M. Groarke, Lisa Graham-Wisener

**Affiliations:** 1 School of Psychology, Queen’s University Belfast, University Road, Belfast, BT7 1NN UK; 2 School of Psychology, University of Galway, University Road, Galway, Ireland

**Keywords:** Caregivers, Caregiver burden, Esophageal neoplasms, Gastrointestinal neoplasms, Pancreas, Stomach, Bile ducts, Gallbladder, Quality of life, Delivery of health care

## Abstract

**Background:**

Upper gastrointestinal cancers (UGICs) are increasingly prevalent. With a poor prognosis and significant longer-term effects, UGICs present significant adjustment challenges for individuals with cancer and their informal caregivers. However, the supportive care needs of these informal caregivers are largely unknown. This systematic review of qualitative studies synthesises and critically evaluates the current evidence base on the experience of informal caregivers of individuals with UGIC.

**Methods:**

A Joanna Briggs Institute systematic review was conducted. Searches were performed in four databases (MEDLINE, PsycINFO, Embase, CINAHL) from database inception to February 2021. Included studies explored experiences of informal caregivers of individuals diagnosed with primary cancer of the oesophagus, stomach, pancreas, bile duct, gallbladder, or liver. Studies were independently screened for eligibility and included studies were appraised for quality by two reviewers. Data were extracted and synthesised using meta-aggregation.

**Results:**

19 papers were included in this review, and 328 findings were extracted. These were aggregated into 16 categories across three findings: (1) UGIC caregiver burden; UGIC caregivers undertake extensive responsibilities, especially around patient diet as digestion is severely impacted by UGICs. (2) Mediators of caregiver burden; The nature of UGICs, characterised by disruptive life changes for caregivers, was identified as a mediator for caregiver burden. (3) Consequences of caregiver burden: UGIC caregivers’ experiences were shaped by unmet needs, a lack of information and a general decline in social interaction.

**Conclusions:**

The findings of this review suggest the need for a cultural shift within health services. Caregiving for UGIC patients is suggested to adversely affect caregivers’ quality of life, similarly to other cancer caregiving populations and therefore they should be better incorporated as co-clients in care-planning and execution by including them in discussions about the patient’s diagnosis, treatment options, and potential side effects.

**Supplementary Information:**

The online version contains supplementary material available at 10.1186/s12913-024-11306-3.

## Background

The National Institute for Clinical Excellence (NICE) [1] define upper gastrointestinal cancers (UGICs) as cancers of the oesophagus, stomach, pancreas, bile duct/gallbladder, or liver. Of all new cancer diagnoses in 2020 globally, 16.6% were UGICs [2]. Incidence of UGICs is increasing in countries under economic transition, and in Western countries due to heightened exposure to certain risk factors [3]. Overall prevalence of UGICs is also expected to rise annually with growing life expectancy and improved diagnostics [4]. Despite this, UGICs still have a uniquely poor prognosis in comparison to other cancer populations [5]. UGICs do not typically benefit from screening programmes and individuals are more likely to present at diagnosis with advanced disease [6]. This is compounded by a high rate of recurrence for individuals able to receive curative treatment [7–9]. As a result, UGICs persistently account for a significant proportion of global cancer deaths; 27.1% in 2020 [2]. Poor prognosis contributes significantly to the heightened disease burden of UGIC, alongside increased utilisation of health services due to the complexity of the treatment trajectory and symptom management [10, 11]. In comparison to other cancer populations, having UGIC is associated with late consultation with palliative care services [12] meaning patients and their families have delayed access, if any, to supportive interventions such as counselling, psycho-education, financial advice and structured family meetings [13].

The supportive care needs of the sizeable population of individuals with UGIC are considerable, with sustained late and longer-term effects. In addition to the common sequalae from cancer diagnosis and treatment, disruption to the digestive system presents problems with swallowing, nausea and keeping food down, a modified diet, extreme changes in weight, chronic pain and living with a stoma [14, 15]. The poor prognosis and longer-term effects present a challenge in adjustment both for the individual with UGIC and their informal caregiver, defined as “close persons” who may be related to the diagnosed individual (siblings, relatives, or spouses) or not (friends, neighbours). A caregiver is anyone identified as such by the patient to provide unpaid ongoing care and support [16]. Examples of challenges for caregivers include learning new practical skills such as managing negative responses to foods, providing a new diet, monitoring weight changes, chronic pain management and stoma management [17, 18]. With biomedical advances leading to a reduction in hospital stay length [19], there is increasing emphasis placed on the role of the UGIC caregiver to provide support to the individual with cancer in the community.

This unique caregiver population face distinct challenges which contribute to caregiver burden which reflects the need for further research into their experiences. For example, due to changes in the diet of the individual with UGIC, the social aspect of dining for both is compromised and can lead to feelings of loneliness, anxiety, and shame [20, 21]. Evidence of caregiver burden is suggested by high levels of anxiety and depression. In caregivers of post-treatment oesophageal cancer patients, 30% of caregivers reported moderate-high levels of anxiety and 10% reported moderate-high levels of depression, alongside a significant fear of recurrence [22]. Research suggests that UGIC caregivers may experience higher levels of psychological distress than the individual with UGIC, and that clinical levels of anxiety and depression may be sustained in the longer-term [22, 23]. However it is worth noting that a lot of the effects of UGIC caregiving acknowledged in the literature are consistent with the general experience of informally providing care and as such there is scope to apply the beneficial practices from other settings (both extra-GI cancer and non-cancer).

It is crucial that we recognise the role of caregivers as co-clients and understand the experiences of this significant caregiver population. Caregivers’ personal experiences are inherently subjective, and due to this subjective nature, a qualitative research approach is optimal [24]. A synthesis of existing qualitative studies will help to establish a knowledge base on the experience of informal caregivers of individuals with UGIC and will help to inform the provision for supportive care. An initial search of PROSPERO, MEDLINE, the Cochrane Database of Systematic Reviews and the Joanna Briggs Institute (JBI) Database of Systematic Reviews and Implementation Reports was conducted and no current or underway systematic reviews on the topic were identified.

### Aim

This qualitative systematic review aims to synthesise the best available evidence on the experiences of informal caregivers supporting individuals diagnosed with UGIC.

## Method

### Design

This systematic review was conducted following the JBI approach to qualitative systematic reviews [25]. A protocol was pre-registered in PROSPERO (registration number CRD42021235354). The systematic review is reported according to the Preferred Reporting Items for Systematic Reviews and Meta-Analyses Protocols (PRISMA-P) statement [26].

### Search strategy

An initial limited search of MEDLINE (Ovid) and PsycINFO (Ovid) was undertaken using the following keywords: Oesophageal cancer OR Stomach cancer OR Gastrointestinal cancer OR pancreas cancer OR gallbladder cancer OR liver cancer AND caregiver AND Qualitative. The text words contained in the titles and abstracts of relevant articles, and the index terms used to describe the articles were used to develop a full search strategy for MEDLINE and adapted for the other databases.

The final search strategy (Additional information 1) was then employed against four databases: MEDLINE (Ovid), PsycINFO (Ovid), Embase (Elsevier) and CINAHL (EBSCOhost). Each database was searched on 12th February 2021.

### Study selection

Following the formal searches, all identified citations were collated and uploaded into Endnote [27] to identify and remove duplicates. Rayyan reference management software [28] was then used by independent two reviewers (DD, MF) to screen titles and abstracts against the eligibility criteria. Potentially relevant articles were retrieved in full and screened against the eligibility criteria by two independent reviewers (DD, MF). Reasons for exclusion of papers at full text review were recorded. Any disagreements that arose between the reviewers at each stage of the selection process was resolved through discussion (DD, MF), or with an additional reviewer (LGW). The reference list and citation list of all eligible articles was searched for additional studies.

### Inclusion and exclusion criteria

#### Population

This review included studies exploring experiences of adults (≥ 18 years of age) who are informal caregivers of individuals diagnosed with UGIC at any stage within the disease process. This included those diagnosed with cancer of the oesophagus, stomach, pancreas, bile duct, gallbladder, or liver [1]. This diagnosis must be the primary cancer site. Studies involving informal caregivers of individuals who had secondary gastrointestinal system metastases were not included.

A caregiver is anyone identified as such by the patient to provide unpaid ongoing care and support [16]. Paid professional caregivers were not included. The caregivers included provided various services, such as practical (providing transport, overseeing meals) or emotional support roles in caring for the patient. Caregivers with any gender or ethnicity were considered for inclusion. Both active and bereaved caregivers were eligible, if discussing their pre-bereavement experience.

Studies which reviewed experiences of multiple groups (e.g., patients, caregivers, healthcare professionals) or multiple cancers beyond the remit of UGIC were included, provided the data pertaining to informal caregivers and UGICs was clearly delineated and could be extracted separately. Where data was hard to distinguish regarding participant-type or cancer-type, the study was only included if at least 50% of the sample size was drawn from the target population.

#### Phenomena of interest

The review included qualitative studies that looked at caregivers’ experiences of caring for an individual with UGIC.

#### Context

Studies for inclusion were based in any geographic location or setting. All care contexts were considered relevant (e.g., primary care, secondary, tertiary, community, or home settings).

### Types of studies

Research studies considered for inclusion were focused on qualitative data including, but not limited to; designs such as phenomenology, grounded theory, ethnography, action research and feminist research. Mixed method studies were considered relevant if data from the qualitative component could be clearly extracted. Only English language studies were included.

Only empirical studies published in peer-reviewed journals were included. There was no restriction on publication year. Systematic reviews were not included, however relevant studies were harvested from them, when relevant. Editorials, opinion papers, case studies and any articles without relevant, original data were excluded, alongside grey literature.

### Quality Appraisal

Subsequently, two independent reviewers (DD, MF) critically appraised the included studies to evaluate the strength of the evidence for methodological quality using the JBI Critical Appraisal Checklist for Qualitative Research [29]. All studies, regardless of the results of their methodological quality, underwent data extraction and synthesis. One of the included studies employed use of free-test questionnaires [30], the robustness of which has been called into question by qualitative researchers as the data generated from these responses is rarely rich enough to provide the necessary strong insights [31]. However, the reviewers felt the robustness of this study was upheld by the fact that the researchers conducted a comprehensive search on existing literature prior to data collection, thus allowing questionnaire findings to be scaffolded onto existing conceptual frameworks.

### Data extraction

Data were extracted using standardized JBI data extraction tool [32] by two independent reviewers (DD, MF). Each undertook data extraction for half of the articles and then checked the other reviewer’s data extraction. The extracted data included specific details about the population, context, study methods and the phenomena of interest relevant to the review objective. Disagreements between the reviewers were resolved through discussion. Four authors of papers were contacted to request missing or additional data for clarification mainly regarding breakdown of participant populations by cancer type of which no new information arose.

A finding is defined by the JBI as “a verbatim extract of the author’s analytic interpretation accompanied by either a participant voice, or fieldwork observations or other data.” [33, p40]. Findings were identified through repeated reading of the text, and extraction of findings included any distinct analytic observation reported by authors with an accompanying illustration (Additional information 2).

### Data synthesis

Each finding was identified by an alphanumeric code (e.g., A1, A2, B1, etc.). Each letter corresponded to a study and each number to a unique finding. The progressive numbers indicate the order of the findings within the original article. Each finding was rated with one of three levels of credibility as per the ConQual system [34]:

1. Unequivocal - findings accompanied by an illustration that is beyond reasonable doubt and therefore not open to challenge.
2. Credible - findings accompanied by an illustration lacking clear association with it and therefore open to challenge.
3. Not Supported - findings are not supported by the data.

Qualitative research findings were pooled with the meta-aggregation approach and captured in a Microsoft Excel spreadsheet [33]. Findings were aggregated by assembling the findings and categorizing these findings based on similarity in meaning, then labelling the categories accordingly. Categories were then synthesised to produce a comprehensive set of synthesized findings. Two reviewers (DD, MF) repeatedly read the findings and developed a set of categories. To assess the quality and confidence of each qualitative finding synthesised within this review, authors utilised the ConQual system (Additional information 3), a tool used to assign ratings of confidence in synthesised qualitative research findings [34]. Only unequivocal and credible findings were included in the synthesis.

## Results

**Fig. 1:**
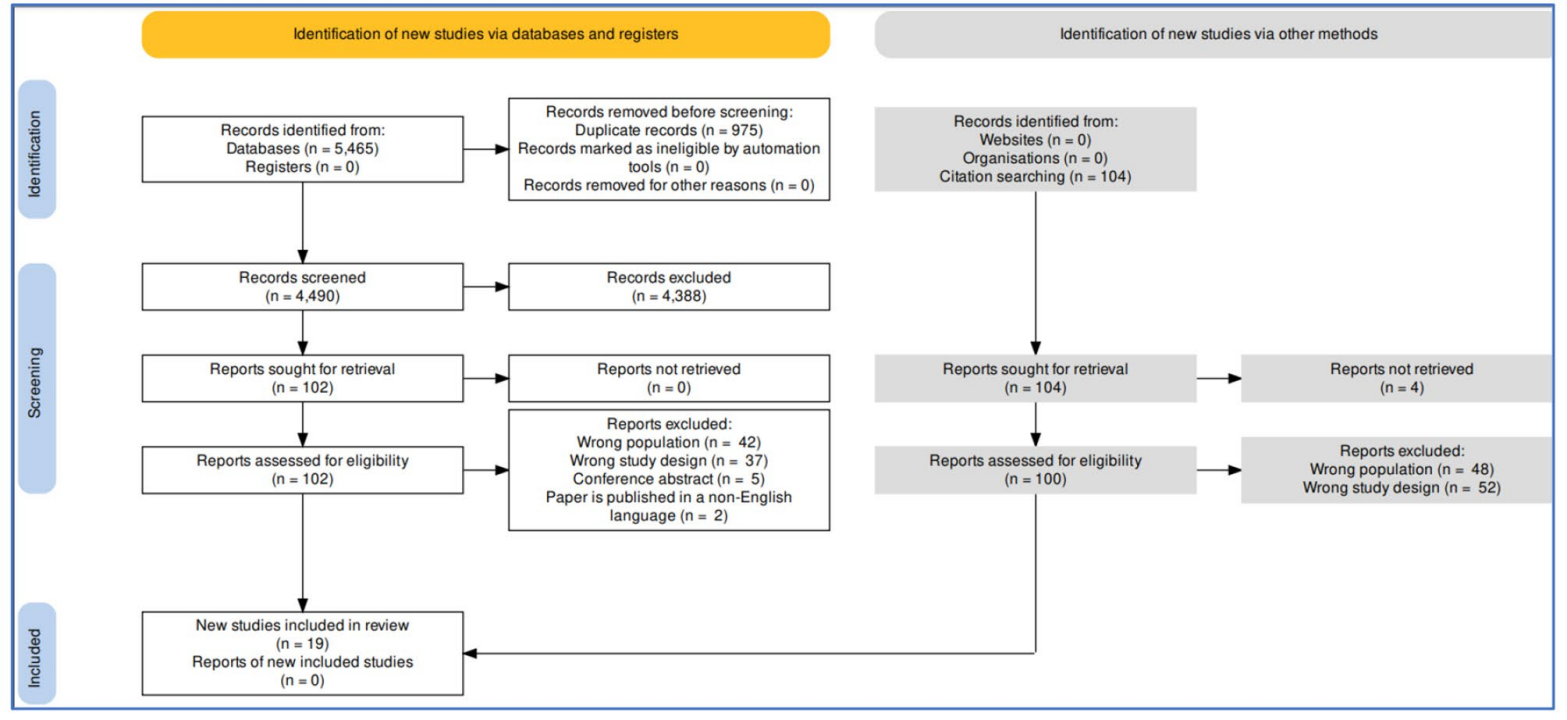
PRISMA flowchart of study selection process

The combined database searches yielded 5465 records. After removing duplicates and screening studies against eligibility criteria (Fig. 1), the review included 19 studies [18, 30, 34–51]. Additional information 4 displays the characteristics of the 19 included studies.

### Description of included studies

All included studies were published between 2004 and 2021. Most commonly, studies focused on caregivers of individuals with oesophageal cancer (*N* = 7), or pancreatic (*N* = 7), including one study of pancreatic and bile duct cancer. Other studies included caregivers of individuals with liver cancer (*N* = 2), gastric cancer (*N* = 1) and the gastrointestinal tract generally (*N* = 2). Geographically, studies were conducted in eight regions. The largest group (*N* = 6) were conducted in the US [35, 37–41], followed by Denmark (*N* = 3) [42–44]. Most samples included a variety of within-family caregivers (*N* = 13), generally spouses/partners, children, and siblings. Others (*N* = 3) looked specifically at spouses and three did not specify the caregiver-patient relationship. Most studies included a semi-structured interview format (*N* = 12), others used focus groups (*N* = 4), secondary analysis of existing data (*N* = 2) or questionnaires (*N* = 1).

### Quality of included studies

The JBI Critical Appraisal Checklist [25] was used to establish the quality of the research. The included studies were generally of good quality, with all 19 papers achieving at least 60% across the ten JBI quality assessment criteria (Additional information 5). Within the JBI checklist there are five questions assessing study dependability, where the studies performed at a lower satisfactory level. Of the included papers, two achieved a 5/5 score on dependability questions, seven achieved 4/5, nine scored 3/5 and one scored 2/5. Only 26% of studies could adequately locate the researcher(s) culturally or theoretically and only 37% of papers addressed the influence of the researcher on the research and vice-versa. Conversely, nearly all papers adequately addressed the research methodology’s congruity on objectives, data collection, data representation and analysis.

### Meta-aggregation findings

**Fig. 2:**
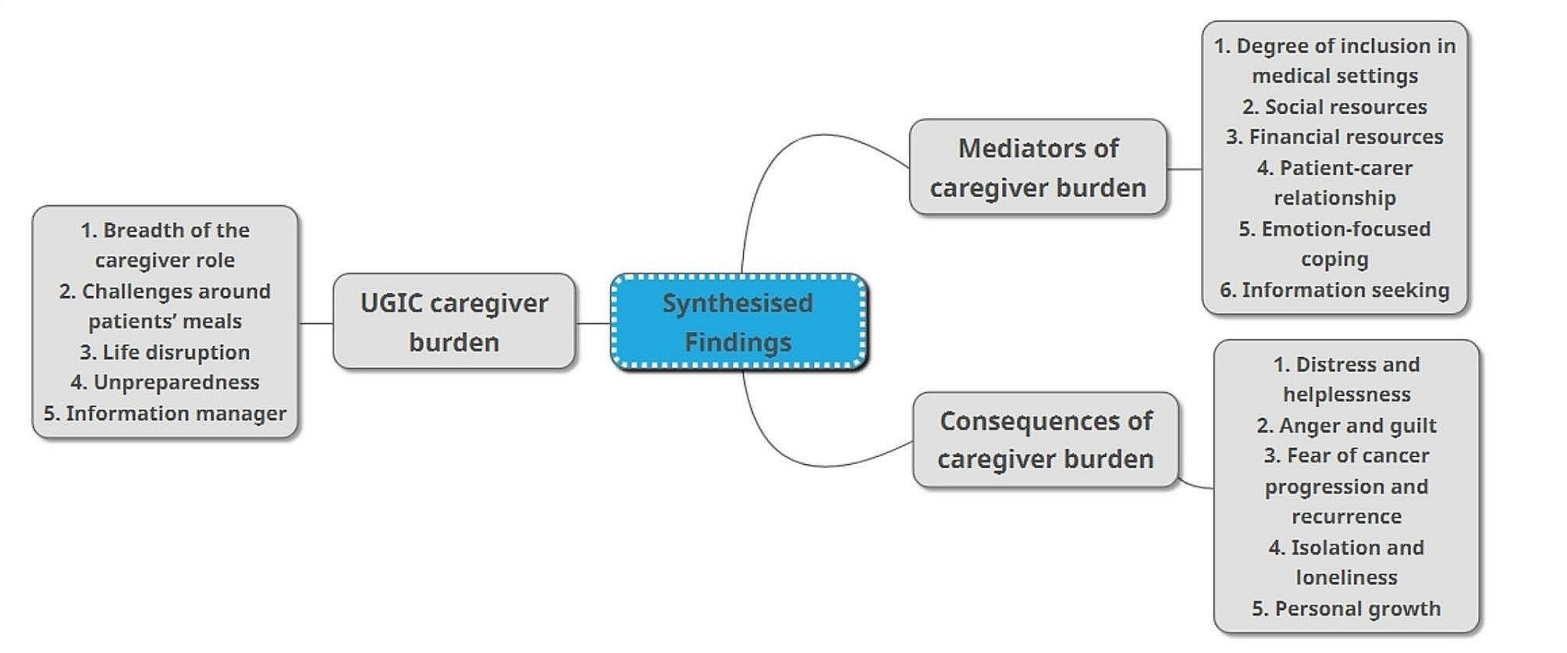
Structural arrangement of categories and synthesised findings

Across the 19 studies, 328 supported findings were extracted, of which 239 were unequivocal and 89 were credible. Findings could be aligned into 23 categories with unique core meanings, which were then synthesised into three findings: (1) UGIC caregiver burden; (2) Mediators of caregiver burden; (3) Consequences of caregiver burden (Additional information 6). Figure 2 outlines how the categories relate to the overarching synthesised findings. To remain grounded in the data, the actual participants’ words are used throughout the narrative and double quotation marks illustrate a direct caregiver quote. References given after a quotation links the quote to the study as outlined in Additional information 2.

### Synthesised finding 1: UGIC caregiver burden

As caregivers began supporting those with UGIC, they faced numerous challenges to adjustment. This largely stemmed from efforts to integrate a broad and complex caregiving role within their existing routine. Difficulties such as disruption to daily routines and meals impacted caregivers’ psychological wellbeing. Caregivers were often unprepared for this life disruption, leading them to seek out information from which to learn and distribute to others.

#### Categories

#### 1. Breadth of the caregiver role

The extent of responsibilities on UGIC caregivers was perceived as broad and complex, with an ‘all encompassing’ focus on patient outcomes. UGIC caregivers ‘assume different roles’ [42].

*“The food thing is omnipresent. We have been told that he is not allowed to have further weight loss (K23).*

Specific responsibilities included working around reduced appetite and oral intake; monitoring physical signs e.g., patient weight; perioperative management such as care of surgical wounds and organising medical appointments and treatments.

*“We’d have to keep. . .going with all the medical appointments and surgery and treatment”* (B11).

#### 2. Challenges around patients’ meals

Treatment for and progression of UGIC severely impacts the patient’s relationship with food; with diet quantity and content at times significantly altered. Adaption for the caregiver involved learning about dietary modifications and management of digestive symptoms such as dysphagia. Several studies found that the new dietary restrictions were a source of worry for caregivers regarding the patients’ weight [44–47]. The social importance of food was a common theme throughout the included studies, with interruption to established social norms perceived as distressing. Mealtimes are considered a ‘unifying family ritual’ [49], but when mealtimes constantly serve to remind caregivers of their responsibility of monitoring, they became a potential source of distress.

*“I can’t get Bernard out of the small meals. . I have to ring him every day from work to tell him to eat”* (A7).

#### 3. Life disruption

UGIC was experienced as coming unexpectedly into caregivers’ lives, intruding on their existing routines, for instance, as working professionals or parents. Caregivers described their responsibilities as time and energy-consuming. This conflict caused caregivers to feel a loss of control [44]. Caregiving responsibilities for UGICs demanded commitment over a long-time frame, impacting caregivers’ employability and their ‘own social life’ [35]:

*“It’s changed my daily routine. It totally disrupted my life. I have to rearrange a lot of things such as my kids*,* my work*,* and getting help for my house cleaning”* (J4).

#### 4. Unpreparedness

Caregivers expressed being ill-equipped and unqualified to manage the needs of the UGIC patient. Caregivers reported feeling out of their depth, partially attributed to the lack of available support, relating to patients’ medical requirements:

*“I went*,* ‘You’re not supposed to call 911? What am I supposed to do? What if he just dies right here?’ I mean*,* it seems they should have somebody say*,* ’OK*,* if he’s with you*,* then here’s the procedure…[The nurse] gave me really no support about what to do”* (R21).

Caregivers sometimes felt misled about the extent of their new responsibilities, as while the patient was cared for in hospital by medical staff, they could not gauge what caregiving at home would involve.

*“I wish they would have talked to me about it as well… it was a bit of a shock. …but the next morning it all dawned on me that I had just replaced a whole team”* (E10).

#### 5. Information manager

Caregivers perceived a key responsibility was to make executive decisions in the dissemination of information, for instance symptomatology, treatment plans and prognosis. Caregivers felt they were the ‘conduit’ [18] through which medical details were communicated to members of the extended social circle, a time-consuming role where they spent *“hours on the phone telling everyone what is happening”* (I32).

The caregivers also viewed their role as giving healthcare providers (HCPs) valuable insight into how the patient was coping outside of the medical setting:

*“[describing a discussion during a clinical consultation*,* contradicting the patient] It is not correct that you almost eat as usual. You are eating food of more liquid substance than you usually do and your drinks are high-protein”* (C1).

### Synthesised finding 2: mediators of caregiver burden

While supporting patients with UGIC, caregivers are exposed to mediators which could increase or reduce caregiver burden, including their use of coping strategies, financial and social resources, and their caregiving context. For instance, higher levels of social support helped alleviate some caregiver burden. Similarly, how excluded a caregiver felt in the medical setting influenced the burden experienced.

#### Categories

#### 1. Degree of inclusion in medical settings

Many studies reported that caregivers perceived they are often kept at a distance in medical settings, increasing caregiver burden. Although some caregivers felt this was fitting and chose to take a ‘subordinate position’ [44], others struggled with a sense of exclusion, which commonly left unresolved questions:

*“…my husband could ask questions*,* but I didn’t have the space to ask questions*,* not unless my husband allowed it”* (K39).

In such cases, caregivers relied on HCPs’ judgement. Caregivers described only being ‘seen’ if they actively called attention to themselves [41]. Caregivers experienced being left out of important decisions.

Caregivers expressed wanting to ask questions without the patient present but felt they had no opportunity to directly communicate with HCPs. This pervasive, default invisibility was perceived as disempowering:

*“No health professionals involved me in this decision”* (K38).

#### 2. Social resources

The degree and quality of support received by caregivers varied and shaped their overall caring experience. The support network is especially beneficial for normalisation of caregivers’ experiences, providing hope and reducing feelings of isolation.

*“it was only when I came here that I started talking to people … it was just like a breath of fresh air. . this dumping syndrome*,* he [the patient] wasn’t the only one”* (A10).

Support could be from spiritual groups (*“I have a lot of people that stand behind me…”* (B19), empathetic HCPs (*“It’s easier to talk with a nurse when it concerns important questions. You may receive quite good and reassuring answers”* (H22) or peers who have undergone a similar caregiving experience, and therefore could reliably address and empathise with caregivers’ challenges.

#### 3. Financial resources

Caregivers reported financial pressure as they had to consider the dyad’s financial situation while one or both members may not be able to work. Providing full-time care was a drain on caregivers’ resources, time, and money. Caregivers struggled with financial planning for the future in the face of prognostic ambiguity.

*“We talked about if we should stay on at the house or sell it”* (K6).

There were additional pressures on dyads living in countries where utilisation of private health services is the norm.

*“Now my grandmother is sick and I can understand how high is the cost of the disease”* (D5).

#### 4. Patient-caregiver relationship

The caregiving experience was shaped by the inter-dyad relationship. Some caregivers reported having an emotionally distant relationship with the patient before the diagnosis which led to poor attachment during the cancer trajectory. Others reported a decline in the relationship quality due to cancer-related pressures.

*“When I got upset*,* I would say to my husband*,* ‘You got cancer because you didn’t listen to me! You deserve it!”* (F35).

Others noted a shift within the relationship, transitioning from ‘caregiver’ to ‘curer’ or from a spousal role to a parental one [45] especially where the caregiver was actively involved in delivering treatment:

*“Sometimes I felt like a mother talking to a child: ‘Remember to do this and that’ ”* (K29).

Caregivers experienced reciprocal suffering when seeing the patient suffer, especially if an established close relationship existed:

*“up when the patient is up and down when the patient is down”* (I21).

#### 5. Emotion-focused coping

The cancer experience was perceived to result in significant distress for caregivers. To address this challenge, caregivers engaged in positive emotion-focused coping strategies to directly regulate distress. Many caregivers reported trying to maintain positive thinking. One participant recalled using humour:

*“Sometimes you can’t believe what happens and the only thing you can do is laugh”* (I41).

Maintaining a positive outlook was perceived to involve *“looking for the good in every situation”* and by being selective about what news caregivers received through ‘denial’ and *“choosing what to hear”* (I44). Conversely, another study described positivity as an open-minded reflection on the conflict between current suffering and spiritual beliefs [38]. Caregivers described how formally addressing their feelings through therapy was also helpful.

Individuals were limited in their opportunity for emotional expression. Caregivers described hiding their own negative thoughts from the patient and took practical measures to divert the patient’s attention by doing *“normal things like [going] for a drive and [having] visits from our children and grandchildren”* (C15).

#### 6. Information seeking

Caregivers perceived challenges around a lack of information from HCPs regarding UGIC’s pathology and related management options. The experiences of caregivers included difficulties in accessing information.

*“We have little information in these areas. When we go to the physician’s office for treatment*,* the doctor is too busy to give us information in this regard and he merely visits the patients. When we see that nobody could survive from such diseases*,* we get worried more”* (D9).

Caregivers addressed the information challenge by persistently seeking information relating to the disease itself, namely cancer-related symptomatology, prognosis, and treatment options (including alternative therapies). Caregivers referred to sources like medically knowledgeable peers, the internet and print (e.g., encyclopaedias). HCPs were trusted for honest information, with their word choices and body language carefully analysed:

*“When my husband and I visit the doctor together*,* you see when he opens the door that there is no good news today”* (H6).

Caregivers were especially empowered when they could differentiate between symptoms due to disease progression and treatment-related adverse effects.

### Synthesised finding 3: consequences of caregiver burden

There were consequences of caregiver burden such as feelings of helplessness, distress, anger, guilt and a strong fear of losing the patient. Conversely, there was potential for positive outcomes as caregivers experienced growth and feelings of hope.

#### Categories

#### 1. Distress and helplessness

When recounting the most involved phase of providing care, active treatment, many caregivers reported experiencing heightened distress. One caregiver perceived gastric cancer a ‘death sentence’ [49], and seeing the patient struggle with the effects of disease and treatments an unbearably ‘*challenging experience’* [40]. This distress also affected children with one spouse noting their child’s *“grades dropped disastrously during his first term”* (H14).

Helplessness originates from a lack of control over the disease progression. A particular source of distress were the delays along the cancer trajectory, especially at diagnosis due to the ambiguous presentation of UGICs and lack of control over symptom management.

*“It is distressing seeing him in pain all the time”* (E6).

#### 2. Anger and guilt

Caregivers experienced a sense of guilt and anger because they perceived stigma from society towards certain cancers. Others may assume that the diagnosis was caused by the patient’s behaviours and therefore indirectly the caregiver may also have been involved. A few studies described this judgement from society towards the patient, with caregivers fearing that others would see the diagnosis as a justified fate:

*“You know*,* when you say cirrhosis of the liver*,* they think*,* ‘Oh*,* you drank yourself’”* (R7).

Caregivers also harboured anger at being forced to take on caregiving responsibilities, describing they had *“been dealt a bad hand”* (I39); however, they felt guilty for feeling this way.

#### 3. Fear of cancer progression and recurrence

Due to the unpredictability of UGICs, caregivers described living in constant dread of the patient’s health declining, and the potential for disease progression or recurrence:

*“I am not sure I am going to like the answers I get. Maybe it is better not to know so very much but to do like the ostrich*,* to bury your head in the sand and hope for the best and keep your fingers crossed”* (H41).

Caregivers were fearful of any new physical or psychological symptoms in patients, especially weight-loss, as caregivers saw this as a marker of recurrence. Further, caregivers feared the cancer would progress to a terminal stage which meant they were afraid of the means through which the bereavement would occur and their own subsequent reaction.

*“the fear of not being sure of how it’s going to happen and how I’m going to react…I’m afraid of losing him”* (L1).

The high mortality associated with most UGICs caused several caregivers to experience acceptance, with the realisation of the long-term impact of their loved one’s cancer and possibility of bereavement.

*“The possibility is there for one of us dying quickly”* (K5).

#### 4. Isolation and loneliness

Caregivers commonly reported experiencing isolation within their unique role, feeling unable to share their anxieties. As patients were burdened already, caregivers did not want to unload their own worries on to the patient.

*“And I had nobody to talk to…There was just nobody. I couldn’t let myself down*,* my guard down and I found the isolation terrible”* (A3).

Loneliness was not only an ongoing concern, but a future threat as spousal caregivers relayed their fear of life post-bereavement.

#### 5. Personal growth

Caregivers reflected that they saw the experience of caregiving as a catalyst for personal change, resulting in positive outcomes such as personal growth and appreciation for life, individually and within the relationship. Caregivers recounted that this unexpected, immense challenge had given them ‘*new perspectives about life’* [35]. Couples got to spend time together that they would not have had otherwise which led to an improved quality of relationship.

*“We’ll talk three or four times a month. Where 10 years ago it might be 6 months or 10 months you know between phone calls”* (B14).

## Discussion

The current study presents the first comprehensive synthesis of qualitative research on the experiences of caregivers of individuals with UGICs. This review is the first to systematically identify and synthesise the current evidence base on the experiences of informal caregivers of individuals with UGIC. Given the emergence of this prominent caregiver population, this review contributes to advancing cancer caregiver literature as a whole, an important area of study recognised by individuals with cancer, their family and healthcare professionals [52]. The review included 19 studies, presented synthesised findings, and identified aspects of caregiving experiences that UGIC caregivers have in common with other cancer caregivers, and aspects more distinct to UGICs. UGIC caregivers experience significant challenges contributing to high levels of burden which are mediated by social, psychological, and practical resources, as well as aspects of health service delivery. The consequences of caregiver burden are primarily negative, including distress, anger, fear, and loneliness.

Caregivers of UGIC patients experienced burden due to the breath and complexity of their role for which they felt unprepared. Caring involved incorporating novel skills into existing responsibilities, causing significant life disruption. Caregivers perceive burden in providing multifaceted care with demands that shift along the illness trajectory. For example, in the beginning caregivers felt it necessary to partake in provision of care, and due to UGIC treatment and disease progression, many responsibilities evolved to monitor and maximise physical health, such as diligent weight monitoring and meal preparation [45–47]. These findings align to the general cancer caregiver literature [53], with caregivers recognised in having steep initial learning curves to rapidly acquire skills to provide care. Only one of the 19 studies evaluated data over an extended period [45]. An extended review is needed to map supportive care resources available across the disease path and longitudinal studies tracking UGIC caregiver support needs across the illness trajectory is warranted.

One of the most reported findings in this review was informal caregivers’ continuous search for information related to their role. Many struggle to satisfy their informational needs at different stages of the disease trajectory contributing to caregiver burden. This corresponds with systematic review findings of Wang et al. [54] that informational needs were the most common unmet need of informal caregivers. To begin addressing this need, caregivers could be signposted to existing sources of general caregiver support information and interventions, such as Cancer Caring Coping [55, 56]. These supports could be used to develop informational resources tailored for UGIC caregivers. A core information set has been developed to aid HCPs at consultation with UGIC patients, to ensure key information is being delivered [57, 58] and now the focus of improving patient-carer education should be raising awareness of this key information toolkit to HCPs who commonly interact with this population. A similar approach could be utilised by identifying informational needs of UGIC caregivers at consultations and developing standardised information points delivered by HCPs to caregivers within those consultations. There is also potential to expand the pool of reliable sources of information to individuals outside of the HCP cohort, such as peer networks or psychologists in providing longitudinal support without necessarily adding to the cost burden required for the development of additional personnel and resources.

This review found caregivers experienced exclusion in the medical setting, suggesting enhanced communication between HCPs and caregivers could improve caregivers’ experience. Indeed, a qualitative study by Reblin et al. [59] identified communication within health services as a key driver for improving cancer caregiver support. One potential avenue to bridge the gap between HCPs and patient-caregiver dyad is incorporating better the clinical nurse specialist (CNS) [60] as these professionals can be a key contact for bi-directional communication between HCPs and caregivers. That is, caregivers support and help the clinical team to understand the patient’s progress and through this process HCPs acknowledge and include caregivers in the patient’s care. However, the current issue of under resourcing in cancer nursing would need to be addressed as it presently limits the amount of CNS time available to support caregivers [61].

One review finding specific to UGIC caregiver burden was the challenge around preparing meals. Taleghani and colleagues [62] mirror this, highlighting gastric caregivers experienced inadequate education in managing patient’s dietary requirements appropriately, resulting in feeling inefficient, uncomfortable, and fearful. Dietician-led interventions are typically patient focused [63–65]. However, this review highlights an opportunity for HCPs to include caregivers in dietician-led interventions as many caregivers assume responsibility over meal preparation and grocery shopping. The challenge around meals also has social consequences as meals are important social settings. Changes in eating behaviours can lead to both dyad members feeling isolated and lonely [18, 66]. Loneliness is prevalent among people living with cancer and is influenced by cancer-specific and non-cancer specific risk factors, such as lack of social support [67]. There is less of an understanding of loneliness among UGIC caregivers compared to general cancer caregivers [68]. This is of concern as negative physical and mental health impacts of loneliness are well-established [69, 70]. Peer support is the most used intervention to reduce caregivers’ loneliness, with strategies of psychoeducation and emotional support featuring prominently [71]. Research is needed to identify risk and protective factors for loneliness among UGIC caregivers.

In addition to loneliness, distress and negative affect were identified as consequences of UGIC caregiver burden. There is evidence of heightened distress and reduced physical and mental health among UGIC caregivers relative to UGIC patients [72, 73]. This review also found that caregivers engage in emotion-focused strategies to cope with their caregiving role. A review by Teixeira et al. [74] found that among cancer caregivers, emotion-focused coping was related to higher distress, whereas problem-focused coping was related to better adjustment and reduced burden. There is a need to develop targeted theory-based psychosocial interventions for this caregiver group. The Transactional Theory of Stress and Coping (TTSC) framework could be utilised to understand how mediating processes specific to coping strategies influence distress and negative affect among UGIC caregivers [75–77], similar to how Bowan et al. [78] used a Baltes and Baltes [79] coping framework to develop interventions for cancer patients’ families. Candidate interventions could involve problem-solving and coping skills training [80, 81], which could in turn ameliorate the negative consequences of caregiver burden. If effective with UGIC caregivers, such interventions could be extended to all caregivers as part of a standard care pathway. This review recommends further research to develop an understanding of adjustment in UGIC caregivers.

In contrast to the many negative consequences described by informal caregivers, there were a small group of findings which indicated some positive outcomes. These findings align with a review of the positive aspects of caregiving, which reported improved relationship quality, reward, fulfilment, and personal growth [82]. The review concluded that positive aspects of caregiving are interconnected and suggested, in addition to interventions reducing negative burden, that interventions could be developed to enhance positive outcomes, such as personal growth. Tedeschi and Calhoun’s Transformational Model (TM) [83] proposes that potentially traumatic stressors, such as caring for an individual diagnosed with cancer, cause a disruption in one’s worldview triggering attempts to make meaning in response to the stressor. Cognitive disruptions also lead to distress, which in turn can act as a catalyst for post-traumatic growth (PTG). Studies have found that caregivers of people with advanced cancer and early-stage breast cancer experience PTG in relation to their caregiving role [84, 85], and that PTG was positively associated with greater social support and perceived hope [86]. Additional research is needed to understand how the challenging UGIC caregiver role may facilitate growth and help the caregiver adjust to their role.

### Study limitations

The current systematic review has several strengths. Firstly, it followed an internationally recognised methodology (JBI) for the conduct of qualitative systematic reviews. This helped ensure methodological approach rigour and subsequently, confidence in findings should they be used to inform policy and practice. There are however several limitations. Although studies in the review are generally of good quality, only 19 studies were identified. Indeed, the UK Less Survivable Cancers Taskforce [87] advocates for more research focused on cancers with low life expectancy, two-thirds of which are UGICs. This lack of research into UGICs extends to the evidence on caregivers. Synthesised findings are therefore based on a small number of studies, largely conducted in the US and Denmark. Within the studies, caregivers of individuals with oesophageal and pancreatic cancer were well represented. However, there were a dearth of studies focused on caregivers’ experiences with gallbladder, or stomach cancer, alongside multiple studies exploring caregivers’ experiences related to dysphagia and malabsorption but fewer exploring jaundice. Therefore, more primary qualitative research is necessary to understand experiences of all UGIC caregiver populations.

### Clinical implications

Of relevance for clinical practice was the finding that caregivers often felt excluded in medical settings, increasing caregiver burden. Caregivers should be seen as co-clients along with patients in the medical setting. This is very much in line with the priorities of care within palliative healthcare settings. Since the palliative care approach seeks to addresses the physical, psychological, cultural, social, and spiritual needs [88] of both individuals with life-limiting and chronic illnesses like cancer and their support networks, early referral to palliative care services could be particularly beneficial for caregivers as their needs are formally and expertly acknowledged and thus help alleviate the burden identified for informal caregivers in this study.

HCPs have an opportunity to give caregivers reliable, specific, and up-to-date information, pitched at the right level to reassure but not overwhelm. Morris and Thomas [89] mirror this suggestion and highlight its importance, as there is potential for tension in information exchange due to HCP’s lack of formal acknowledgement of caregivers. Clinical guidance and policy could be updated to include recognition of caregivers as co-clients, and with caregiver training, could formally be part of the patient support team. This could help meet the caregivers’ needs, especially post-diagnosis. On an institutional level, caregivers may be more recognised within their role if acknowledged formally, for example in NICE [1] guidelines for UGICs. In understanding the considerable role caregivers undertake supporting the care of UGIC patients outside of the healthcare system, policymakers and HCPs need to improve support for caregivers which will in turn reduce the burden on health services.

## Conclusion

The aim of this qualitative systematic review was to synthesize evidence about the experiences of UGIC caregivers and has found that caregivers face significant challenges leading to caregiver burden which negatively impacts adjustment. Due to the nature of UGICs, caregivers experienced unique challenges such as how best to manage disruptions to mealtimes and how to monitor surrogate markers of patient health, such as weight. UGICs are a medically complex and evolving chronic condition and caregivers struggle to gain information. This review found that caregiver burden was impacted by feeling excluded in medical settings which could be improved with better communication between HCPs, patients, and their caregivers. There is a lack of data relating to the experiences of certain UGIC caregivers (e.g., gallbladder, stomach) in comparison to others (e.g., oesophageal), as well as a lack of understanding on how to manage the impact of caregiving for these types of cancer, thus providing directions for future research.

### Electronic supplementary material

Below is the link to the electronic supplementary material.

**Supplementary Material 1: Additional file 1** Search Strategy

**Supplementary Material 2: Additional file 2** Findings illustrations table

**Supplementary Material 3: Additional file 3** ConQual table

**Supplementary Material 4: Additional file 4** Characteristics of studies

**Supplementary Material 5: Additional file 5** Methodological assessment

**Supplementary Material 6: Additional file 6** Meta-synthesis

## Data Availability

No datasets were generated or analysed during the current study.
