# Supplementary material for: Experiences of informal caregivers supporting individuals with upper gastrointestinal cancers: a systematic review
Source: BMC Health Serv Res. 2024 Aug 14;24:932. doi: 10.1186/s12913-024-11306-3 (PMC11325824; doi:10.1186/s12913-024-11306-3)
Supplement: Supplementary file 1 — Supplementary Material 1: Additional file 1 Search Strategy [file 12913_2024_11306_MOESM1_ESM.docx]

**Additional file 1 - Search Strategy**

Database: Ovid MEDLINE(R) ALL <1946 to April 06, 2020>

| **#** | **Searches** | **Results** |
| --- | --- | --- |
| 1 | Esophageal Neoplasms/ | 51298 |
| 2 | Stomach Neoplasms/ | 98062 |
| 3 | Gastrointestinal Neoplasms/ | 18257 |
| 4 | Digestive system cancer/ | 3637 |
| 5 | Pancreatic Neoplasms/ | 74939 |
| 6 | Liver Neoplasms/ | 151355 |
| 7 | Cholangiocarcinoma/ | 9107 |
| 8 | Bile Duct Neoplasms/ | 14931 |
| 9 | "esophageal tumo*" or "esophageal neoplasm*" or "esophageal cancer*" or "esophageal carcinoma*" or "oesophageal tumo*" or "oesophageal neoplasm*" or "oesophageal cancer*" or "oesophageal carcinoma*" or "esophago-gastric tumo*" or "esophago-gastric neoplasm*" or "esophago-gastric cancer*" or "esophago-gastric carcinoma*" or "gastro-*esophagal junction tumo*" or "gastro-*esophagal junction neoplasm*" or "gastro-*esophagal junction cancer*" or "gastro-*esophagal junction carcinoma*" or "gastric tumo*" or "gastric neoplasm*" or "gastric cancer*" or "gastric carcinoma*" or "stomach tumo*" or "stomach neoplasm*" or "stomach cancer*" or "stomach carcinoma" or "hepatic tumo*" or "hepatic neoplasm*" or "hepatic cancer*" or "hepatic carcinoma*" or "liver tumo*" or "liver neoplasm*" or "liver cancer*" or "liver carcinoma*" or "duodenal tumo*" or "duodenal neoplasm*" or "duodenal cancer*" or "duodenal carcinoma*" or "biliary tumo*" or "biliary neoplasm*" or "biliary cancer*" or "biliary carcinoma*" or "gallbladder tumo*" or "gallbladder neoplasm*" or "gallbladder cancer*" or "gallbladder carcinoma*" or "pancrea* tumo*" or "pancrea* neoplasm*" or "pancrea* cancer*" or "pancrea* carcinoma*" or "gastro-intestinal tumo*" or "gastro-intestinal neoplasm*" or "gastro-intestinal cancer*" or "gastro-intestinal carcinoma*" or "thorax tumo*" or "thorax neoplasm*" or "thorax cancer*" or "thorax carcinoma" | 448569 |
| 10 | Caregivers/ | 38418 |
| 11 | caregiver*/ or carer*/ or care-giver*/ or parent*/ or father*/ or mother*/ or visitor*/ or friend*/ or "patient* interpersonal relation*"/ or family/ or families/ or "family characteristic*"/ or "family relation*"/ or "intergenerational relation*"/ or spous*/ or husband*/ or wife/ or wives/ or partner*/ or neighbor*/ or neighbour*/ or "next of kin"/ or non-professional/ or "non professional"/ or volunteer*/ or relatives | 146664 |
| 12 | qualitative* or "focus group*" or "grounded theory" or (grounded adj theory) or (framework adj analysis) or IPA or "narrative analysis" or "thematic analysis" or (thematic adj analysis) or (constant adj comparison) or TA or interview* or "mixed method*" or mixed-method or "narrative approach" or ethnograph or journal or diary or phenomenol* or "discourse analysis" or "content analysis" or "group discussion" or "audio record" or audiorecord or lifeworld* or "life world*" or life-world* or "constant comparative" or "constant comparison" or "biographical method" or "open-ended" or "open ended" or experience | 30045227 |
| 13 | 1 or 2 or 3 or 4 or 5 or 6 or 7 or 8 or 9 | 473880 |
| 12 | 8 or 9 | 175461 |
| 13 | 10 and 11 and 12 | 1203 |
| 14 | exp animals/ not humans.sh. | 4782806 |
| 15 | 13 not 14 | 1203 |
| 16 | limit 15 to English language | 1073 |
