# Supplementary material for: Experiences of informal caregivers supporting individuals with upper gastrointestinal cancers: a systematic review
Source: BMC Health Serv Res. 2024 Aug 14;24:932. doi: 10.1186/s12913-024-11306-3 (PMC11325824; doi:10.1186/s12913-024-11306-3)
Supplement: Supplementary file 2 — Supplementary Material 2: Additional file 2 Findings illustrations table [file 12913_2024_11306_MOESM2_ESM.docx]

**Additional file 2 - Findings/illustration table**

Evidence: [U] Unequivocal – [C] Credible – Unsupported

Illustration with page number

| **Article Reference:** | **(A) McCorry *et al* (2009)** |
| --- | --- |
| Finding (1) | Responsibility for protecting the patient and their family from distress [withhold information] [U] |
| Illustration | “They also told me that if he hadn’t had the operation, if they hadn’t got him back to surgery that night it would have been too late . . . he is not aware of that, as a matter of fact nobody else in the family is aware of that because I think a secret’s best kept if you really keep it to yourself.” (p11) |
| Finding (2) | Needing to be strong for those around them [U] |
| Illustration | “I felt em, I had to be strong for the whole family because I would be a strong person anyway, but they were all looking to me and I couldn’t let the side down.” (p11) |
| Finding (3) | Carer’s feelings of isolation [U] |
| Illustration | “And I had nobody to talk to. I was nursing my father with cancer, my sister had just died, I had cancer, John had cancer. There was just nobody. I couldn’t let myself down, my guard down and I found the isolation terrible.” (p11) |
| Finding (4) | Elevated levels of distress, often resulting in altered sleeping and eating patterns and reduced self-care of their own health problems |
| Illustration | No illustration (unsupported) |
| Finding (5) | Burden of responsibility for patient’s recovery [U] |
| Illustration | “You were trying to get him to eat, trying to get him to take his tablets and I was getting the brunt of everything. And that was the worst . . . and it was so hard you know, and I used to have to go out of the room because I started crying” (p11) |
| Finding (6) | Carer was also a conduit who provided explanations to family and friends [C] |
| Illustration | “I had to take the guy away to the side and I says “look, would you mind coming back and removing the plate and not saying anything because” – well, I told him the situation.” (p11) |
| Finding (7) | Carers' representations of food and eating were heavily emotionally laden and they still represented recovery in terms of the ability to eat larger quantities. [C] |
| Illustration | “I can’t get Bernard out of the small meals . . . I have to ring him every day from work to tell him to eat but his eating has got a bit better and he’s put on a bit of weight.” (p12) |
| Finding (8) | Weight loss, dumping, or feeling unwell as indicators of disease recurrence [U] |
| Illustration | “Every time that he would not feel well or would have the dumping syndrome, I keep wondering is it back?” (p12) |
| Finding (9) | Varied experiences of support from health professionals |
| Illustration | No illustration (unsupported) |
| Finding (10) | Recognized the value of peer support, especially for normalization of experiences reducing feelings of isolation, and a source of hope [U] |
| Illustration | “it was only when I came here that I started talking to people . . . the first lifeline we had was here [the support group] . . . it was just like a breath of fresh air . . . and things that Brian had, this dumping syndrome, he wasn’t the only one” (p12) |
| **Article Reference:** | **(B) Petrin *et al* (2009)** |
| Finding (1) | Initial shock and disbelief upon learning the news [diagnosis] [U] |
| Illustration | I think right after the diagnosis it was very difficult. It was a shock. Offspring, living relative) (p283) |
| Finding (2) | Period of denial and devastation [C] |
| Illustration | My father and I had a close relationship and [the news] was devastating. Offspring, living relative) (p283) |
| Finding (3) | Fear of the disease and what the future might bring coupled with an overall sadness [U] |
| Illustration | [I was] feeling scared and upset and . . . I guess sad too. (Offspring, deceased relative) (p283) |
| Finding (4) | Ascribed these emotions not only to themselves but to other family members as well [fear and sadness] |
| Illustration | No illustration (unsupported) |
| Finding (5) | Pancreatic cancer diagnosis as having a significant impact on their lives. [C] |
| Illustration | My father and I had a close relationship and [the news] was devastating. (Offspring, living relative) (p283) |
| Finding (6) | Information seeking was one of the most common coping-related themes. [U] |
| Illustration | I needed to get more information I think was the big thing. I needed to find out . . . so exactly what does this mean? (p283) |
| Finding (7) | Wanted to know how long their loved one could be expected to survive, how severe the symptoms would be, and what treatments were available [U] |
| Illustration | I was in school and had access to PubMed. . . .I [read] a lot of the journal articles myself so I had kind of a good idea of what the research said about  life expectancies and different treatments. (Offspring, living relative) (p284) |
| Finding (8) | Feeling that things were happening quickly and that time was of the essence [U] |
| Illustration | he was in the hospital and he had his treatments and he had his surgery and everything just went—seemed to go so fast. (Sibling, living relative) (p284) |
| Finding (9) | Family members felt responsible for helping the patient by taking care of scheduling and attending doctor’s appointments and treatments, activities seemed to be to take one’s mind off of the emotional handling of the illness. [U] |
| Illustration | No one really had time to stop and to pause to do any, you know, reflection. . . . We’d have to keep . . . going with all the medical appointments and surgery and treatment. (Offspring, living relative) (p284) |
| Finding (10) | Need to manage aspects of his own life [C] |
| Illustration | I had to take care of my children. Try and straighten out my financial matters and household and . . . have to work of course. (Offspring, living relative) (p284) |
| Finding (11) | Feeling of being able to contribute in some way was helpful. [U] |
| Illustration | No one really had time to stop and to pause to do any, you know, reflection. . . . We’d have to keep . . .going with all the medical appointments and surgery and treatment. (Offspring, living relative) (p284) |
| Finding (12) | Make more time for the ill relative. [U] |
| Illustration | Jjust being with him was the big thing . . . just our normal stuff, had Saturday dinners and played games and watched movies and stuff. Just tried to make it as normal as possible. I tried to go see him more. (Offspring, deceased relative) (p284) |
| Finding (13) | If visiting was not an option, because of a physical distance, for example, this was a source of significant stress. [U] |
| Illustration | I attribute it to . . . I guess having a lot, you know, like being so far away and not being able to just drop in and see him whenever I want to and still going to work every day and coming home and being stressed out and eating and also I attribute it to the antidepressants. (Sibling, living relative) (p284) |
| Finding (14) | The increased time spent benefited the relationships for those with surviving relatives. [U] |
| Illustration | We’ll talk three or four times a month. Where 10 years ago it might be 6 months or 10 months you know between phone calls. (Sibling, living relative) (p284) |
| Finding (15) | Many participants, however willing to discuss emotions with the interviewer, confessed to having hidden their feelings during the time of crisis [C] |
| Illustration | Sometimes you have to put aside your own feelings and emotions and . . . concentrate on the one that’s sick and just be there for him. (Sibling, deceased relative) (p285) |
| Finding (16) | The degree to which individuals felt they received support and sources of that support varied. [C] |
| Illustration | Kind of the nature of our family to—you can argue on the outside but when something’s important you just kind of hang together and take care of it. (Offspring, living relative) (p285) |
| Finding (17) | the importance of communicating with family members, often with the ill individual him- or herself. [U] |
| Illustration | Kind of the nature of our family to—you can argue on the outside but when something’s important you just kind of hang together and take care of it. (Offspring, living relative) (p285) |
| Finding (18) | took comfort in being able to talk to friends, particularly those who had a similar experience of dealing with an ill family member [C] |
| Illustration | I have a lot of good friends and . . . I have a lot of people that stand behind me. . . . |
| Finding (19) | others sought solace in the church, both in attending church services as well as engaging in religious observance in private. [C] |
| Illustration | I have a strong faith and . . . I have a lot of good friends and . . . I have a lot of people that stand behind me. . . . I think I did okay, you know. (Sibling, living relative) (p285) |
| Finding (20) | Those who coped in this way found it to be helpful in sorting out their feelings and in dealing with them. [Therapy included counselling and in one instance, a course of antidepressant therapy] [C] |
| Illustration | [The counselor] just helped me kind of deal with the fact that, um, it’s not something that’s catchy, you know, that I don’t have to sit around and keep myself from my children in bubbles in hopes that we don’t get it. (Sibling, living relative) (p285) |
| Finding (21) | others were hampered by a lack of family communication or more formal types of social support. [U] |
| Illustration | I felt like I couldn’t open up and talk to [my husband] about it because . . . I don’t think it was because he didn’t want to hear it. I think he just didn’t want to see me so upset. (Sibling, living relative) (p285) |
| Finding (22) | Family dynamics, conveying either implicitly or explicitly that whatever underlying foundation had existed prior to the diagnosis remained intact. [U] |
| Illustration | How families deal with things is how they’re going to deal with this. Kind of almost predetermined in their relationship than it is something that’s pancreatic specific, I guess. (Offspring, deceased relative) (p285) |
| Finding (23) | Those families that were initially distant maintained this pattern as well. [U] |
| Illustration | We are all individuals and I think we kind of reacted as individuals. (Sibling, living relative) (p285) |
| Finding (24) | some participants coming from families that were distant harbored resentment over being burdened with caring for the ill family member. [U] |
| Illustration | I feel like, you know, there should be more of the share of the responsibility instead of myself running the whole show and dealing with it, emotionally and financially. So, I feel there is a great sense of resentment. (Offspring, living relative) (p286) |
| Finding (25) | concern about their own future health and that of other family members. [U] |
| Illustration | [I wonder] what is the probability of my having cancer and going through this and fear of pain. And fear of, you know, if I, if this is genetic that it would pass down to my son. (Offspring, living relative) (p286) |
| Finding (26) | many individuals resolved to positively change their behavior [U] |
| Illustration | [My sister’s diagnosis] made me take notice and do something that I hadn’t been able to do in a long time. I smoked cigarettes for 40 years and I quit. (Sibling, living relative) (p286) |
| Finding (27) | One individual, however, expressed the opposite Sentiment [to positively change their behaviour] [U] |
| Illustration | Her brother, now stricken with pancreatic cancer, had always been extremely health conscious. She concluded fatalistically, “It doesn’t matter how you take care of your body” (Sibling, living relative). (p286) |
| Finding (28) | newfound awareness of their own mortality and a new appreciation for the value of their own lives [C] |
| Illustration | I just don’t put up with as much, you know. Life’s too short. If I don’t want to do something, I’m not going to do it. If I don’t want to go somewhere, I’m not going to go. (Sibling, living relative) (p286) |
| Finding (29) | less worry with the passage of time [surviving relative [U] |
| Illustration | But as time goes by and he’s getting along so well, you kind of relax. (Parent, living relative) (p286) |
| Finding (30) | disbelieving, even in the face of evidence that the patient’s cancer was in remission [U] |
| Illustration | I’m feeling a little better about the whole thing. I’m sure it’ll come back, but, anyhow, he’s doing pretty good and does a lot of the stuff that he used to do. (Offspring, living relative) (p286) |
| Finding (31) | Family members of patients who succumbed to pancreatic cancer described a challenging experience. [U] |
| Illustration | I had a real hard time because I just didn’t want to give up. I didn’twant to relinquish the fact that she was going to pass on so I was constantly looking and reading and doing whatever I could trying to find something . . . that we could try that . . . might help her. (Sibling, deceased relative) |
| Finding (32) | Individuals saw pancreatic cancer as having an impact on their lives for many years to come. [C] |
| Illustration | know when the time comes, it’s going to be bad for me. (Parent, living relative) |
| **Article Reference:** | **(C) Larsen *et al* (2021)** |
| Finding (1) | relatives were generally present in the patients’ consultations with health professionals; Relatives could add important information in consultation [U] |
| Illustration | The wife looks at her husband and says, ‘It is not correct that you almost eat as usual. You are eating food of more liquid substance than you usually do and your drinks are high-protein.’ (p280) |
| Finding (2) | relatives often put into words their view on how the patient managed everyday life [U] |
| Illustration | The wife looks at her husband and says, ‘It is not correct that you almost eat as usual. You are eating food of more liquid substance than you usually do and your drinks are high-protein.’ (Field notes P19) (p280) |
| Finding (3) | relatives were not always invited to be an active part of the consultation by the health professionals [U] |
| Illustration | The wife is close to choosing a chair but is told by the doctor, that this chair belongs to the patients. (Field notes P2) (p280) |
| Finding (4) | observations showed that relatives often did not have an established role in consultations but were merely looked upon as an appendage to the patients leading to relatives were positioned on the sideline and not anchored in consultations. [U] |
| Illustration | The wife is close to choosing a chair but is told by the doctor, that this chair belongs to the patients. (Field notes P2) (p280) |
| Finding (5) | relatives had an awareness of the severity of the disease [U] |
| Illustration | Well I am aware that the speed in diagnosing means that the sooner start of treatment the better. (Interview P1) (p281) |
| Finding (6) | did not get time to reflect about the new life situation [U] |
| Illustration | You can write off your chance of growing accustomed. to the new situation. (Interview P10) (p281) |
| Finding (7) | the speed in diagnosing made them feel behind in the schedule. [U] |
| Illustration | Well I am aware that the speed in diagnosing means that the sooner start of treatment the better. (Interview P1) (p281) |
| Finding (8) | feel obliged to inspire confidence in the patient regarding future and put up a facade. [U] |
| Illustration | Being a relative is a strain on you, because you feel you need to put on a facade and look happy. (Interview P4) (p281) |
| Finding (9) | suppressed their own anxiety and doubt about the treatment and future [U] |
| Illustration | I think it was worse for me than my husband. He was in his usual way. The day we got the diagnosis I was totally confused and didn´t know what to do. . . I am afraid that my husband won’t make it. I wake up every night with my jumble of thoughts. (Interview P17) (p281) |
| Finding (10) | felt an obligation to possess strength not only for themselves but also for the patient |
| Illustration | No illustration (unsupported) |
| Finding (11) | Struggled with thoughts and possible worries about the future. [U] |
| Illustration | I think it was worse for me than my husband. He was in his usual way. The day we got the diagnosis I was totally confused and didn´t know what to do. . . I am afraid that my husband won’t make it. I wake up every night with my jumble of thoughts. (Interview P17) (p281) |
| Finding (12) | Relatives felt their daily lives collapsing and they lost control. [C] |
| Illustration | I said sell all of it. I cannot manage dealing with it by myself. Your whole life is turned upside down. (Interview P18) (p281) |
| Finding (13) | fears about their economic situation and had a need to establish an overview of the situation [C] |
| Illustration | I thought I was going to work, but I felt so miserable and couldn’t do anything but cry. (Interview P18) (p281) |
| Finding (14) | struggled to keep a sense of perspective and felt obliged to be the anchor in the uncertainty regarding the future and possible treatment. [U] |
| Illustration | Well I try being there and supporting my husband as much as I can. I try not to be too negative in my thoughts and get overwhelmed by the situation. I thought I was going to work, but I felt so miserable and couldn’t do anything but cry. (Interview P18) (p281) |
| Finding (15) | create a refuge from the disease and balance everyday life. [U] |
| Illustration | The disease cannot be present all the time. It is there, but we need to do normal things like go for a drive and have visits from our children and grandchildren. (Interview P1) (p281) |
| Finding (16) | Relatives had a constant awareness about the disease, and going on with ordinary life was a way to handle the difficult situation [unsupported] |
| Illustration | No illustration (unsupported) |
| Finding (17) | could not handle the uncertainty on a long-term basis but needed to take it day by day. [U] |
| Illustration | We need to take one day at a time and see what happens. (Interview P16) |
| Finding (18) | maintaining everyday life was important and helped them feel capable and active. |
| Illustration | No illustration (unsupported) |
| Finding (19) | diagnosis was a challenge for the relatives, and they struggled to changed living conditions |
| Illustration | No illustration (unsupported) |
| Finding (20) | Knowing there was the possibility of treatment was a comfort and a relief. [U] |
| Illustration | When we were told that the cancer hadn’t spread and it looked treatable, then the knot in my stomach disappeared. That information was comforting. It was like a light at the end of the tunnel. (Interview P18) (p282) |
| Finding (21) | should not be part of decisions, and it was natural that health professionals did not invite them to participate. [U] |
| Illustration | In my opinion it is my husband who needs to play a leading role. He is the one who makes the decision in the end. I am comfortable being in the background. (Interview P5) (p282) |
| Finding (22) | relatives took a subordinate part in consultations, as they considered the consultation to be owned by the patient. [U] |
| Illustration | He is the one who makes the decision in the end. I am comfortable being in the back-ground. (Interview P5) (p282) |
| Finding (23) | might have questions themselves, which they did not always get the permission to ask. [U] |
| Illustration | It is my husband who has the disease but I also need to know stuff. We don’t always agree on which questions to ask. (Interview P14) (p282) |
| Finding (24) | Relatives and patients were not always in agreement about which questions to ask, which made it difficult for relatives; they were left with unanswered questions. [U] |
| Illustration | It is my husband who has the disease but I also need to know stuff. We don’t always agree on which questions to ask. (Interview P14) (p282) |
| Finding (25) | amount of information they received from health professionals was large, but they also acknowledged the need for an overview of the treatment pathway [U] |
| Illustration | We receive so much information, and you can think, come on, it’s a lot to digest, but it gives you a sense of security, knowing what to expect. (Interview P5) (p282) |
| Finding (26) | Knowledge of what to expect and how to manage the effects of treatment made relatives feel more at ease with the whole situation. [C] |
| Illustration | It is nice that the information is straightforward and nothing is sugar-coated. (Interview P7) (p282) |
| Finding (27) | It was important that health professionals were honest in their communications. [U] |
| Illustration | It is nice that the information is straight forward and nothing is sugar-coated. (Interview P7) (p282) |
| Finding (28) | they were unfamiliar with the treatment and that it could be difficult to know which questions to ask. [U] |
| Illustration | Well, it is so easy, telling patients, if they have any questions. What on earth should they ask about? They have no ideas. (Interview P10) (p282) |
| Finding (28) | sometimes they chose not to ask questions about the future as they relied on the authority of health professionals. [U] |
| Illustration | I wouldn’t say we have been involved. We have merely been informed about the best treatment pathway and accepted the proposed plan. That’s fine. We are not the experts. (Interview P5) (p282) |
| **Article Reference:** | **(D) Morowatisharifabad, *et al* (2019)** |
| Finding (1) | Fear of isolation and loneliness. [C] |
| Illustration | “my wife gets very quiet after the disease, and does not participant in our family gatherings.” [P8]. (p959) |
| Finding (2) | Fear of disease disclosure [U] |
| Illustration | I was very stressed after my father disease but I did not even let my own family know about it. I told myself that my mom was ill and had blood pressure. If I said, it would hurt my family and the situation would get worse.”[P15]. (p959) |
| Finding (3) | Fear of loss of social status. [C] |
| Illustration | Due to this disease, the positions of the roles that you have had will change and you should be a burden to others and as a result, you will be worthless to others. [P59] (p960) |
| Finding (4) | fear of difficulty of treatment [U] - Some participants feared from long-term treatment of the disease and therapeutic follow-ups. |
| Illustration | it was very difficult for me because my mother did not have any desire to eat during chemotherapy. My mother had severe diarrhea and vomiting after each session of chemotherapy and she felt fatigue ...”[P16]. (p960) |
| Finding (5) | fear of financial problems and burdens. [U] |
| Illustration | Now my grandmother is sick and I can understand how high is the cost of the disease.” (p960) |
| Finding (6) | Fear of impatience to treatment [U] |
| Illustration | “I wish I never get these diseases. I do not have the patience to take my drugs when I have cold; let alone having cancer, which needs constant treatment and follow ups. I certainly can not bear it....” [P7]. (p960) |
| Finding (7) | following cancer diagnosis was mental changes that caused mental distress in participants. [U] |
| Illustration | “I myself saw that one of my friends lost her control and died after having heard the name of her illness... I just have one child in this world and I live far away from my family and my relatives. I do not want to think about this and I cannot imagine that I get this disease one day...” [P3]. (p960) |
| Finding (8) | Fear of cancer. Involved experiencing different stresses in relation to this disease [esophageal cancer] [U] |
| Illustration | “Doctors say that these treatments increase life expectancy of patients, but they doubt about complete remission ... I’m very afraid of these diseases because of death. I am afraid of knowing that I am going to die sooner because of my disease.” [P7]. (p961) |
| Finding (9) | lack of information about the disease. [U] |
| Illustration | “We have little information in these areas. When we go to the physician’s office for treatment, the doctor is too busy to give us information in this regard and he merely visits the patients. When we see that nobody could survive from such diseases, we get worried more.” [P22]. (p961) |
| Finding (10) | Fear of mental reactions. [U] |
| Illustration | “I was so disappointed after my mother’s illness and I went to the psychiatrist. I sometimes feel I catch my mother’s disease. I am eating well, but then I feel it gets stuck in my throat...” [P19]. (p961) |
| Finding (11) | Fear of physical reactions. [U] |
| Illustration | “I was very afraid. Even when I was asleep, I dreamed about my mother’s disease and conditions. I jumped out of bed crying and I afraid more. I could not sleep because my uncle died of this disease. I always had a nightmare....” [P19]. |
| Finding (12) | Fear of emotional changes [U] |
| Illustration | “When I think of cancer, I worried. I prefer generally to think about my disease as a fungus or gland since I have a lot of wishes and I do not want to focus on the disease...” [P7]. (p961) |
| Finding (13) | The fear of appearance changes and the effect of different drugs and treatments on the appearance of patient caused distress in the patient family. [U] |
| Illustration | “My mother got worse during her treatment and she was losing her hair. I was very afraid that the treatment would change the appearance of an individual...” [P7]. (p961) |
| Finding (14) | fear of weight loss [C] |
| Illustration | “When my father eats, he throws up. He has weight loss and feels tiredness and fatigue…” [P13]. |
| **Article Reference:** | **(E) Shaw *et al* (2020)** |
| Finding (1) | This series of interviews highlighted the emotional burden of caring for upper GI cancer patients post-surgery. Across interviews, family caregivers described their stress and anxiety. [U] |
| Illustration | It really battered me around…, it really wears you down. I couldn’t cope I had to get out…I’m tired of it. Tired of the pain but thankful[ly] he’s not so dependent on me now (004) (p751) |
| Finding (2) | This distress was exacerbated when the caregiver and patient were yet to discuss the extent of disease and prognosis with the surgeon |
| Illustration | No illustration (unsupported) |
| Finding (3) | [This distress was exacerbated when] where the patient was in considerable distress or pain. |
| Illustration | No illustration (unsupported) |
| Finding (4) | participants reported that they were physically and emotionally exhausted [U] |
| Illustration | It really battered me around..., it really wears you down. I couldn’t cope I had to get out...I’m tired of it. (004) (p751) |
| Finding (5) | Some family caregivers were still actively involved in the caregiver role while others were trying to get back to a sense of normality. [C] |
| Illustration | I’m getting tired. Cause I need a holiday...I’m just a bit tired (015) (p751) |
| Finding (6) | Family caregivers described being distressed by the cancer diagnosis and the impact of surgery on the patient. [C] |
| Illustration | It is distressing seeing him in pain all the time. (004) (p751) |
| Finding (7) | understanding of the surgery but were unprepared for the level of patient care required. [U] |
| Illustration | It is distressing seeing him in pain all the time. He’s not silent in pain, he’s moaning. So you know that really gets to you because you can’t help (004) (p751) |
| Finding (8) | family caregivers perceived that they lacked the knowledge to meet all the patient’s needs and were fearful of providing inadequate care. [C] |
| Illustration | I don’t think they gave us enough information about how to look after him when we got home… [such as] these are the symptoms you are going to experience and how to cope with them…I didn’t get anything (005) (p752) |
| Finding (9) | Family caregivers also wanted information about what the implications of the diagnosis meant for longer term patient care. [unsupported] |
| Illustration | No illustration (unsupported) |
| Finding (10) | lack of specific information was exacerbated when, prior to discharge, family caregivers were not present during discussions with the treating team [U] |
| Illustration | I wish they would have talked to me about it as well… it was a bit of a shock. I happily took him home …but the next morning it all dawned on me that I had just replaced a whole team (004) (p752) |
| Finding (11) | Family caregivers of non-English-speaking patients experienced a greater sense of isolation as they perceived that language difficulties limited access to information and support. |
| Illustration | I’ve had to do some research so I’ve been left to answer a lot of his questions because my parents wouldn’t really get the English (012) (p752) |
| Finding (12) | Information related to patient nutrition was of particular concern to family caregivers at both time points and contributed to family caregiver distress. [U] |
| Illustration | I suffered terrible stress because he lost so much weight and the main thing was that he was supposed to eat and he wouldn’t (004) (p752) |
| Finding (13) | focus on patient care, often to the exclusion of self-care, was a major contributing factor to family caregivers’ experience of stress. [C] |
| Illustration | ‘I didn’t talk to psychologists or anything. It was going to my bedroom at night you know crying’ (017) (p753) |
| Finding (14) | study interview was the first time they had reflected on their own feelings [U] |
| Illustration | ‘I think it’s been therapeutic for me…to talk through some of the things that you even find difficult to talk to your wife about because you’re sort of scared’ (002) (p753) |
| Finding (15) | the focus on the patient was all encompassing. These participants adopted sole responsibility for patient care. [U] |
| Illustration | I dropped my life…if I was working or had my own family my mum wouldn’t be here today… (017) (p753) |
| Finding (16) | given up activities to support the patient, including work outside the home [U] |
| Illustration | I dropped my life…if I was working or had my own family my mum wouldn’t be here today… (017) |
| Finding (17) | these participants reported that there was little time to consider their own need for support as they were trying to meet the needs of others. [C] |
| Illustration | I dropped my life...if I was working or had my own family my mum wouldn’t be here today (017) |
| Finding (18) | Others reported having to balance their care for the patient with other pre-existing caring and work responsibilities. [U] |
| Illustration | I’m a young mum with four children so I tell you it was very, very difficult… running backwards and forwards…Dad is blind so he’s difficult too because you not only have to look after one but two people (020) (p753) |
| Finding (19) | family caregivers did not perceive themselves as Carers. Providing care was viewed as a natural extension of the family relationship [U] |
| Illustration | well people ask me and I say I’m caring for my sick husband at home. So that’s the language I use. I’m looking after him, I’m supporting him umm….Well it’s something we’re going through, I know that. So when you’re the carer you’re sitting outside, it’s not for you (004) I look after him because he is my husband and I love him (005) (p753) |
| Finding (20) | Perceived as meeting patient physical needs, and the multifaceted responsibility of providing support to the patient. [role] [C] |
| Illustration | after the surgery, caregiver but…[now] the support needs go to a little bit more to…his psychological needs.(002) (p753) |
| Finding (21) | Lack of information regarding the availability of financial and com-munity support resulted in additional stress for family care-givers; little awareness regarding the role of the hospital social work team in facilitating support and few had met with a social worker. [U] |
| Illustration | The stressfulness is not knowing where to go for help(017) (p753) |
| Finding (22) | A pre-existing relationship with a GP was a major determinant of ongoing practical support. [C] |
| Illustration | Several family caregivers reported consulting with their GP about simple medical concerns (e.g. routine wound care) only to be referred to hospital emergency departments for these minor issues, resulting in long delays in accessing care. Attendance at emergency was reported to be stressful for both the patient and the family caregiver. |
| **Article Reference:** | **(F) Yi *et al* (2004)** |
| Finding (1) | The gastric cancer couples considered diagnosis of gastric cancer as receiving “a death sentence.” [U] |
| Illustration | “If you were diagnosed as cancer, you would lose your mind. You would go mad and think, ‘Oh my god! I’m going to die!’” (p629) |
| Finding (2) | Fear of getting worse or recurrence of the cancer |
| Illustration | Unsupported |
| Finding (3) | Could not rely on health professionals for their health anymore [after treatment was over] [C] |
| Illustration | “After talking my last anti-cancer drugs, I felt like I was abandoned in a desert. I hadn’t had a the faintest idea how to live on my own.” (p629) |
| Finding (4) | Taking charge of their health |
| Illustration | Unsupported |
| Finding (5) | Given information regarding diets, they found it extremely hard to follow the regimens. |
| Illustration | Unsupported |
| Finding (6) | GI problems further made the couples frightened, because they regarded them as indicators of recurrence of cancer [U] |
| Illustration | “The thought of recurrence of cancer always hovers in the back of my mind. If he says that he feels something inside his belly, all of a sudden my heart stops.” (p629) |
| Finding (7) | The spouses devoted their entire energy into cooking, providing the best freshest food to the survivors at every meal [U] |
| Illustration | “Cure comes from devotion. There is a saying, ‘Sincerity moves heaven.’” (p629) |
| Finding (8) | Felt that if something happened to the survivor, it would be their fault. |
| Illustration | No illustration (unsupported) |
| Finding (9) | Husbands of female survivors did not take this kind of responsibility but arranged for a person to prepare meals for some time |
| Illustration | No illustration (unsupported) |
| Finding (10) | This devotion to cooking had made female spouses physically drained because most survivors expected to eat newly cooked food at every meal [U] |
| Illustration | “I am living in the kitchen, 24 hours a day…Even now [eight months after surgery] he doesn’t eat for lunch what was prepared for breakfast…he seems to think I am made of steel!” (p629) |
| Finding (11) | Took from several months to several years for the couples to adjust to the new diets….spouses were able to prepare the meals without being drained of physical energy. |
| Illustration | No illustration (unsupported) |
| Finding (12) | When preparing folk medicine the spouses became a curer rather than a carer for the survivors, thinking that their partners’ live relied totally on them [U] |
| Illustration | “To save my wife, I got her every [folk] medicine people said were good, even those that cost a lot of money. Because I wanted to do everything I could for her.” (p630) |
| Finding (13) | It became a way to avoid guilt or reproach from the survivors or other family members if the survivors got worse. [U] |
| Illustration | “I said to my husband, you can’t say that I did not try hard enough.” (p630) |
| Finding (14) | Spouses focused more on “reinforcing physical strength” to get the survivors well [C] |
| Illustration | “eat up as if they were taking medicine, even if it’s hard” |
| Finding (15) | If the survivors did not eat up as expected, the spouses got very disappointed and blamed them for lacking the will to live |
| Illustration | No illustration (unsupported) |
| Finding (16) | Spouses began to realize that pushing the survivors too hard was not good at all for their physical strength, especially when the survivors experienced Dumping syndrome |
| Illustration | No illustration (unsupported) |
| Finding (17) | Gastric cancer couples sought information, wanting to see their situation more realistically, and thus cope with their situation |
| Illustration | No illustration (unsupported) |
| Finding (18) | Couples wanted to know the most about was the causes of their cancer (in their own terms). They thought by knowing the reason they could correct them and prevent the cancer from recurring |
| Illustration | No illustration (unsupported) |
| Finding (19) | The most common reason they found was unhealthy lifestyles, such as drinking, smoking, and having an irregular diet. |
| Illustration | No illustration (unsupported) |
| Finding (20) | Some couples thought the survivors’ perfectionist personalities or neglecting their religious duties were the reason |
| Illustration | No illustration (unsupported) |
| Finding (21) | Conflicts occurred between the couples, especially when the spouses felt that the survivors were not putting in enough effort. |
| Illustration | No illustration (unsupported) |
| Finding (22) | It made spouses disappointed and frustrated, and in the end some even reproached the survivors for getting the cancer [U] |
| Illustration | “When I got upset, I would say to my husband, ‘You got cancer because you didn’t listen to me! You deserve it!” (p630) |
| Finding (23) | In the cases in which they could not find any causes, they also went through emotional distress because there were no faults to correct |
| Illustration | No illustration (unsupported) |
| Finding (24) | Couples also sought information regarding the progress of the illness |
| Illustration | No illustration (unsupported) |
| Finding (25) | Couples were not satisfied with this information [from health professionals] |
| Illustration | No illustration (unsupported) |
| Finding (26) | Did not want to believe some of the information such as the prognosis that they received |
| Illustration | No illustration (unsupported) |
| Finding (27) | Dissatisfaction made them actively seek details and information involving folk medicine that promised cure...from friends and family members and other cancer survivors. |
| Illustration | No illustration (unsupported) |
| Finding (28) | The spouses tried to help the survivors strengthen their Ki. |
| Illustration | No illustration (unsupported) |
| Finding (29) | They set aside their needs and desires, including their sexual needs |
| Illustration | No illustration (unsupported) |
| Finding (30) | They encouraged them to live not for themselves but for their family members, especially for their children and parents. |
| Illustration | No illustration (unsupported) |
| Finding (31) | They also encouraged them to outlive their parents who were still alive, since in Korea, it is an important virtue as a son or daughter. |
| Illustration | No illustration (unsupported) |
| Finding (32) | Spouses thought that strengthening Ki too much was risky because they could loosen up and become self-indulgent, returning to their pervious unhealthy lifestyles. |
| Illustration | The spouses thought that they just had to absorb their complaints like a sponge [their survivor spouse] [C] |
| Finding (33) | “When my wife complains, I just laugh and say ‘I saved your life, and all I get for it is complaints.’” (p630) |
| Illustration | They acted like this because they thought oppressing the survivors’ anger could result in stress, which could cause recurrence of cancer (Unsupported) |
| Finding (34) | They felt pitiful toward the survivors and also thankful towards them for just being alive |
| Illustration | No illustration (unsupported) |
| Finding (35) | The relationship between the spouses became unstable, even for those who had no marital problems before. They became overly sensitive and annoyed at each other. [C] |
| Illustration | Accepting his frustration and anger was so hard. For every thing I said, he got irritated and emotional for no reason….So it got me to the point where I said things I didn’t really mean. Though I try to understand him, I am a human being too, you know. (p631) |
| Finding (36) | Few couples….knew how to talk back appropriately, and thus managed the anger or emotional distress that the survivors expressed |
| Illustration | No illustration (unsupported) |
| Finding (37) | Support from their spouses was necessary in [helping change survivors’ value systems to accept modest life according to Taosim, Buddhism or Christianity] |
| Illustration | So I told him to be free of it, not to worry about it any more, that a person who almost went to heaven’s door does not have to think about [money]. I said that life itself is the only thing he should think about. (p631) |
| Finding (38) | Spouses began to prepare for the future to come |
| Illustration | No illustration (unsupported) |
| Finding (39) | Were afraid of their partner’s death and subsequent life without a partner [U] |
| Illustration | I am never completely relaxed at all. I suffer…I try to allay my loneliness. When I go to social gatherings, I drink till dawn and have a good time to forget the pain, though the thought of my wife never leaves my mind. I just live on, trying to soothe myself. I nod off after a drink, wake up to see it’s already morning, and go to work. I come home in the evening and eat dinner, watch TV, look at my wife from time to time, go to work again. That’s my life. That’s the way I live. (p631) |
| **Article Reference:** | **(G) Padron *et al* (2018)** |
| Finding (1) | male caregivers derived a sense of control over their lives and environments through concrete problem-solving. [C] |
| Illustration | Caregiver #27 reported redecorating his wife’s office as a way to help her manage her distress and fill her life with beauty, stating: “…what I realized was that it was an environment that could make it better, so I needed to go out there and keep it up” (T1, L46). (p42) |
| Finding (2) | Most caregivers did not report being consciously aware of patients’ high distress levels, possibly indicating that avoidance strategies exercised by patients may help maintain caregivers’ sense of peace, control, and overall self-mastery. |
| Illustration | No illustration (unsupported) |
| Finding (3) | Male caregivers reported benefiting from this form of caregiving [patients were caring for their caregivers despite needing help themselves] |
| Illustration | No illustration (unsupported) |
| **Article Reference:** | **(H) Andreassen *et al* (2005)** |
| Finding (1) | Diagnosis of oesophageal cancer disrupted the family member’s lives |
| Illustration | No illustration (unsupported) |
| Finding (2) | Due to the vague symptoms of oesophageal cancer as well as the patient’s lack of awareness of the disease, family members in this study reported that there was a delay in consulting the physician with the symptom. [U] |
| Illustration | ‘It is clear that if my husband had come to the doctor some years earlier, perhaps he could have undergone surgery. You don’t notice the cancer here (points to the throat) before it has gone too far.’ (p428) |
| Finding (3) | in connection with patients receiving their diagnosis, they experienced a delay in receiving a correct diagnosis [U] |
| Illustration | First, when he got ill, it was by chance that they found out, because my husband first visited the general practitioner, who thought it was a virus infection. (p428) |
| Finding (4) | Negative feelings were expressed in relation to the delay in diagnosis [U] |
| Illustration | ‘Everything was very unpleasant when we received this information, since it seems to me that the doctors in fact were guilty of a mistake’. (p428) |
| Finding (5) | The family members experienced that the physicians tried to counterbalance hope and honesty by using words like ‘cell changes’ (dysplasia) when giving the diagnosis of oesophageal cancer. They interpreted the physicians’ wording with a life-threatening disease. [U] |
| Illustration | I guess we suspected that it was a little more than cell changes. It is just the way they describe it in order not to be too shocking. (p428) |
| Finding (6) | Not only the physicians’ vocabulary but also their body language was interpreted carefully [U] |
| Illustration | You are very receptive when you wait to be informed, and you observe people. When my husband and I visit the doctor together, you see when he opens the door that there is no good news today. (p429) |
| Finding (7) | Family members had not anticipated that the patient could suffer from oesophageal cancer. Thus, when faced with this diagnosis, shock, stress and disbelief were evoked. [U] |
| Illustration | I had no idea about this diagnosis, and that was tremendously bewildering for me. It was a shock that such a life-threatening illness was concealed. (p429) |
| Finding (8) | diagnosis of oesophageal cancer seemed to be more bewildering for the family members than the patients [U] |
| Illustration | ‘We were completely shocked when we found out about it. More shocking for me, I believe, than what it was for my wife. At least it seemed that she took it more naturally than I did’. (p429) |
| Finding (9) | Receiving the diagnosis of oesophageal cancer seemed to be more bewildering for the family members than the patients. [U] |
| Illustration | We were completely shocked when we found out about it. More shocking for me, I believe, than what it was for my wife. (p429) |
| Finding (10) | family members experienced that healthcare professionals focused on the care of the patients, which made the family members feel ‘invisible’. [U] |
| Illustration | The most I have talked about the illness, I have perhaps done today during the interview. We are probably only an appendage. (p429) |
| Finding (11) | It was found that the cancer became intrusive in the family’s life and routines [U] |
| Illustration | ‘We can’t travel . . . we can’t live a normal life’. (p429) |
| Finding (12) | Eating was seen as a unifying family ritual. Thus, when someone in the family was unable to eat due to dysphagia, loss of togetherness and social life was evident. [U] |
| Illustration | Usually, we sit together at supper and talk about the day. Then we share our lives with the entire family. It’s very much a sharing opportunity. However, eating together is no longer associated with pleasure because my husband is not able to eat due to his dysphagia. (p429) |
| Finding (13) | Family members in this study emphasized the importance of including the whole family in the care given, even the children, whatever their level of knowledge or ability to understand are, because the children were aware that a tremendous change had occurred in the family. [C] |
| Illustration | I don’t think anyone has ever asked how old our children are, if they visit school or anything like that. They don’t seem to care that there is a family around the patient and that we in fact have a sixteen-year-old son, who has grown up with this. (p429) |
| Finding (14) | children became anxious and stressed which affected their school life. Moreover, they had to struggle much on their own. [U] |
| Illustration | Our son had his 18th birthday this year. Although he himself says that his mother’s illness doesn’t affect him at all, we have noted that his grades dropped disastrously during his first term. (p430) |
| Finding (15) | Crucial for the family members was that their children should participate in information giving. Participation could facilitate the children’s preparedness. [C] |
| Illustration | I think it would be good to receive joint information, to involve the children, since the parent, who comes home is a little foreign. You can say: ‘One parent left and another one came home who is also a patient at home.’ (p430) |
| Finding (16) | The family members experienced an everyday symptomatic uncertainty and looked for signs for deterioration. [U] |
| Illustration | You know all the time that one day it will get worse. You may receive an answer that it is a metastasis, exactly as we received now. I live constantly with this. (p430) |
| Finding (17) | A prognostic uncertainty is a medical reality in patients with oesophageal cancer [C] |
| Illustration | ‘Since after five years one is considered be out of the danger zone, we can calculate that my husband will in some form be given a clean bill of health, but perhaps not quite be declared healthy’. (p430) |
| Finding (18) | The uncertainty of death and dying pervaded the family members’ thoughts and plans for the future. |
| Illustration | ‘Shall we sell the house or shall we not? Shall we renovate our house or shall we not. Shall I work full time or shall I not?’ ‘Will my husband die tomorrow, or what?’ (p430) |
| Finding (19) | The family members expressed a genetic threat and concerns about the connection between genetics and cancer. [U] |
| Illustration | What worries me most is that the illness will affect the children. If they will get this . . . whether it is hereditary. (p430) |
| Finding (20) | the family members had different strategies for managing uncertainty. [C] |
| Illustration | They used interpersonal sources, i.e. experts (physicians, nurses, patients, social network and kinship) as well as media sources, i.e.(encyclopaedias, hospital produced material, Internet, TV)for medical information about the illness and cancer in general, but also to handle uncertainty. (p431) |
| Finding (21) | the family members entrusted themselves to the experts, i.e. the physicians, who were considered the major source of information [U] |
| Illustration | When you are so close to the experts as we are now, we ought to get the truth directly from the doctor if there is anything we wonder about. We have entrusted ourselves to the experts. (p431) |
| Finding (22) | the family members also felt connected to the nurses who could answer questions of importance and give practical and emotional support. [U] |
| Illustration | It’s easier to talk with a nurse when it concerns important questions. You may receive quite good and reassuring answers. / . . . / You get a feeling of trust when you talk with a nurse. (p431) |
| Finding (23) | the patients themselves were considered experts. [U] |
| Illustration | I haven’t asked anything myself because I knew that my husband would ask everything so minutely himself. I know he would look up everything himself. He has shared his knowledge with me and we have discussed it together. (p431) |
| Finding (24) | Despite knowing that the physicians are able to provide information about diagnosis, prognosis and treatment, the family members did not always turn to them with questions. |
| Illustration | Unsupported |
| Finding (25) | They sometimes thought they could not formulate questions since they did not always know enough in order to ask. |
| Illustration | No illustration (unsupported) |
| Finding (26) | This lead to a feeling of being left out of certain knowledge that perhaps should be of value for understanding the situation. |
| Illustration | No illustration (unsupported) |
| Finding (27) | all of the family members did not want to discuss and ask specific questions with the physician when the patient listened. [U] |
| Illustration | I don’t want to ask the doctor a question, which he has to respond to negatively when my husband is with me. (p431) |
| Finding (28) | not asking questions was due to their lack of medical knowledge about oesophageal cancer. [U] |
| Illustration | You are not enough medically knowledgeable. Therefore, you don’t know what to ask. (p431) |
| Finding (29) | The family members contacted persons in the family’s circle who had specific knowledge of the illness and in whom they felt confidence. [C] |
| Illustration | I trusted the judgements that doctors in our acquaintance circle gave, but not completely, since they are not in the field. They can’t be well read in all areas. (p431) |
| Finding (30) | Concerning oesophageal cancer, they were ignorant and had never heard of the disease. [U] |
| Illustration | I hadn’t heard about that disease. I think you have heard about most of the variations, but not cancer of the oesophagus. (p431) |
| Finding (31) | the family members believed that the image of cancer given in Swedish mass media is that the survival rates are increasing. [U] |
| Illustration | I receive most of the information through the mass media. In that way, I get my information and it is sort of positive, since more and more people pull through. (p431) |
| Finding (32) | The family members looked in encyclopaedias, medical books, material produced by the hospital, and brochures, to gain medical information about the illness and to get an overview of problems related to the illness. [U] |
| Illustration | I have an encyclopaedia at home, which certainly is a bit old. I also have a book for quick medical reference, where I can look up different things in order to be able to read briefly about them. (p432) |
| Finding (33) | Family members did not only seek information in order to gain increased medical knowledge, but also because it gave them the feeling of doing something constructive. [U] |
| Illustration | Seeking information is much more than receiving knowledge, it also includes a feeling of doing something. (p432) |
| Finding (34) | used the Internet mainly to obtain an overview about the illness and illness-related problems as well as about the prognosis of oesophageal cancer. [U] |
| Illustration | I think that the Internet was a great help, since it is difficult to telephone someone and pose relevant questions when I hardly know what I want to find out. Then it is possible that if you receive incorrect information, you can form an opinion later. (p432) |
| Finding (35) | when the family members in this study found out about the poor survival rates, they became shocked and worried. [U] |
| Illustration | The prognosis was so bad. It was so depressing and I started to believe that I would find my husband dead in bed. I got terrified and there was nothing positive at all in the information I read. (p432) |
| Finding (36) | When the family members confronted the physicians with information about the prognosis of oesophageal cancer, they found that their reaction was positive. [U] |
| Illustration | I said to the doctor that I had been on the Net and read about a study where it said that there was a terribly poor prognosis. He said that the information was not really current and that the prognosis is better now. I didn’t go into greater detail. (p432) |
| Finding (37) | During the time following diagnosis, the family members’ information seeking was low. |
| Illustration | No illustration (unsupported) |
| Finding (38) | there was an oscillation between family members’ desire for more information and the avoidance of new information. [U] |
| Illustration | I want to know if the prognosis is terribly poor or if it is about one year. I want to know what will happen. . . . Actually, I really don’t want to know (p432) |
| Finding (39) | On the other hand, knowledge about details relating to the illness could alleviate some of the scariness and unpleasantness. [U] |
| Illustration | Perhaps it isn’t so terrible. Everything you know something about loses its terribleness. (p432) |
| Finding (40) | Seeking information was sometimes considered as an effort for the family members, which demanded a considerable amount of time, courage and energy. [U] |
| Illustration | Certainly I can search for information. That isn’t the problem but the problem is that it takes time. I shall mobilise the courage, the power, the energy . . . (p432) |
| Finding (41) | The family members were also afraid of what they might find. [U] |
| Illustration | am not sure I am going to like the answers I get. Maybe it is better not to know so very much but to do like the ostrich, to bury your head in the sand and hope for the best and keep your fingers crossed. (p433) |
| **Article Reference:** | **(I) Sherman *et al* (2014)** |
| Finding (1) | Symptoms were the sentinel event that initiated the caregiving context and engagement with the health care system, with the hope that symptoms were caused by anything other than cancer. [U] |
| Illustration | He had stomach pains but thought it was because the stress of Workd so he took antacids. But when his eyes started to become yellow, I knew it was something serious and begged him to go see the doctor.’’ |
| Finding (2) | caregivers expressed feeling ‘‘lost in the health care system.’’ [U] |
| Illustration | ‘‘What should we do, who do we turn to, how do we proceed?’’ (p389) |
| Finding (3) | The health care system offered little help in navigating the system, [U] |
| Illustration | ‘‘with no point person to serve as coordinator.’’ (p389) |
| Finding (4) | Family caregivers were shocked by the ‘‘sudden and unexpected’’ diagnosis of advanced pancreatic cancer. [U] |
| Illustration | ‘‘An assumption that life will be long and that a person’s state of health is within their control, dependent on their lifestyle and behaviors’’ was violated. (p390) |
| Finding (5) | If the patient had a healthy lifestyle, it was unfathomable to both patient and caregiver that the symptoms were from a life-limiting terminal disease. [U] |
| Illustration | ‘‘He ran every day, ate well, and we thought he would have a long healthy life and we would live into old age together we were wrong.’’ (p390) |
| Finding (6) | Several participants spoke about how others enhanced or interfered with interactions with health care professionals given their own expectations,  fears, and degree of trust of the health care system. [U] |
| Illustration | ‘‘Everyone in our family has their opinions and own experiences with doctors, so at times it was too much.’’ (p390) |
| Finding (7) | Within the context of the patient/ caregiver relationship, there were varying ages and degrees of emotional closeness dependent on the history of the relationship. [U] |
| Illustration | their mother was ‘‘their best friend’’ or …‘‘the relationship was not close before.’’ (p390) |
| Finding (8) | The family caregiver may be the primary caregiver, who assumed total responsibility on a 24 hours basis. [C] |
| Illustration | ‘‘Above everyone else, mom knows that I have always been the one to help her she can always count on me.’ P390 |
| Finding (9) | Alternatively, a caregiver may assume secondary responsibilities for caregiving, acting as support when the primary caregiver needs relief, or there was a sharing of responsibilities with other family members. [U] |
| Illustration | ‘‘Dad takes care of her (mother) but I call every day and come over a couple of times of week to help with doctor’s appointments or help when she is sick from the treatments. As the older sister, I have always felt it was my responsibility.’’ (p390) |
| Finding (10) | Often, physical proximity to the patient resulted in expectations by patients, caregivers themselves, or extended family that the member in closest physical proximity would assume a direct caregiving role. [U] |
| Illustration | ‘‘Dad takes care of her (mother) but I call every day and come over a couple of times of week to help with doctor’s appointments or help when she is sick from the treatments. As the older sister, I have always felt it was my responsibility.’’ (p390) |
| Finding (11) | Family members who lived farther away often were relieved that they did not have direct responsibility for caregiving but also expressed a sense of lack of control over the situation. |
| Illustration | Unsupported |
| Finding (12) | Family caregivers discussed openly either a willingness to care born out a sense of responsibility or duty or (less often) a sense of ‘‘being forced’’ to assume caregiving responsibilities, with a perception of great personal sacrifice. [C] |
| Illustration | As the older sister, I have always felt it was my responsibility.’’ (p390) |
| Finding (13) | Increased family interaction at a time of heightened anxiety exacerbated preexisting negative family dynamics and issues. |
| Illustration | No illustration (unsupported) |
| Finding (14) | As the patient’s needs increased and the final days approached, with more family members in attendance, family caregivers felt an invasion of their personal space and a lack of privacy. [U]) |
| Illustration | ‘‘With everyone over, there is no private time and no place to retreat.’’ (p390) |
| Finding (15) | Family caregivers also spoke of competing priorities when the patient’s needs were primary; followed by the needs of children, spouses, and others; and compounded by the demands of work. |
| Illustration | No illustration (unsupported) |
| Finding (16) | Caregivers’ self-care was not a priority. [U] |
| Illustration | ‘‘He is the focus of my life I will take care of myself when it’s over.’’ (p390) |
| Finding (17) | lack of clear information and support from health professionals was often an intense source of distress. [C] |
| Illustration | ‘‘Physicians are just making educated guesses and no one really has the answer.’’ |
| Finding (18) | revealing or concealing the diagnosis and the prognosis to patients. (p390) |
| Illustration | ‘‘hope would be taken away if she knew it was cancer but yet she knew something was really wrong.’’ (p390) |
| Finding (19) | caregivers expressed a need to understand the whole process. [U] |
| Illustration | ‘‘You don’t know what to expect or have the bigger picture. Information is important to understanding what to expect.’’ (p390) |
| Finding (20) | the caregiver served as protector of not only the patient but also other family members who could not accept and face the death of someone they love. [U] |
| Illustration | ‘‘we dance around the edges’’ with death looming near but ‘‘having to put on a happy and positive face’’ (p392) |
| Finding (21) | the family caregiver and other family members experienced reciprocal suffering [U] |
| Illustration | ‘‘up when the patient is up and down when the patient is down.’’ (p392) |
| Finding (22) | when caregivers seem to achieve some equilibrium, a new wave of bad news or unmanaged patient symptoms overcomes them |
| Illustration | No illustration (unsupported) |
| Finding (23) | caregivers became extremely distressed by a sense of lack of control and often a sense of failure. [as disease progressed and death takes hold] [U] |
| Illustration | ‘‘I am most stressed now that the effect of cancer is seen on the outsidedher color, her weight loss, and the falling out of her hairdand the worst is that I can’t do anything to change it.’’ (p390) |
| Finding (24) | Caregivers, however, were ‘‘wanting to be helpful’’ and ‘‘take off the pressure,’’ which created a sense of resentment on the part of the patient. [U] |
| Illustration | ‘‘He is trying to protect me and still wants to be in charge so that I am not as stressed but I want to do things for him because he is the one who is sick.’’ (p390) |
| Finding (25) | Caregiver guilt was experienced [U] |
| Illustration | ‘‘pressuring her to continue treatment’’ (p392) |
| Finding (26) | Anticipatory grief was manifested as caregivers want to maintain hope when the patient was ‘‘ready to let go.’ |
| Illustration | No illustration (unsupported) |
| Finding (27) | Maintaining hope also involved believing in miracles. [C] |
| Illustration | ‘‘if we stay positive, positive things will happen.’’ (p392) |
| Finding (28) | for many caregivers, the ‘‘fear of being alone’’ was a driving force for ‘‘holding on.’’ [C] |
| Illustration | ‘‘I don’t know what I’ll do without him’’ (p392) |
| Finding (29) | Caregivers also realized that the losses were incremental loss of companionship, normalcy, physical intimacy, etc. |
| Illustration | No illustration (unsupported) |
| Finding (30) | Many caregivers throughout the illness experience hoped or believed that perhaps they could will them (the patient) into wellness by making sure that the patient ate well, got rest, and maintained a positive attitude. |
| Illustration | No illustration (unsupported) |
| Finding (31) | caregivers mirror the courage of the patient when they are really [falling apart] [C] |
| Illustration | ‘‘falling apart inside.’’ (p392) |
| Finding (32) | Family caregivers were the conduit of medical information to other family members [U] |
| Illustration | ‘‘I spend hours on the phone telling everyone what is happening.’’ (p390) |
| Finding (33) | The caregiver’s own social life became limited [U] |
| Illustration | One caregiver remarked, ‘‘I used to enjoy going to church but now I don’t want to leave him home alone so I am missing my church community.’’ (p390) |
| Finding (34) | Caregivers related that ‘‘friends don’t know what to say’’ or even worse ‘‘just don’t ask’’ and that really hurts [U] |
| Illustration | ‘‘friends don’t know what to say’’ ….‘‘just don’t ask’’ (p393) |
| Finding (35) | caregivers cannot find the time to de-stress and take time out to care for their own physical, emotional, or spiritual needs (unsupported) |
| Illustration | No illustration (unsupported) |
| Finding (36) | Caregivers also spoke about losing pleasure in life and expressed concern that their work suffered. |
| Illustration | Unsupported |
| Finding (37) | Caregivers’ identity and meaning and purpose in life were intimately tied to the relationship with the patient [C] |
| Illustration | ‘‘I will have no one to care for I won’t know what to do with myself.’’ (p391) |
| Finding (38) | Part of the angst expressed by caregivers was in second guessing themselves as to whether they had made the right decision. |
| Illustration | No illustration (unsupported) |
| Finding (39) | For some, anger surfaced [U] |
| Illustration | ‘‘have been dealt a bad hand,’’ (p393) |
| Finding (40) | resent the injustices of life, (unsupported) |
| Illustration | No illustration (unsupported) |
| Finding (41) | Several participants cited laugher and humor as ways of coping. [U] |
| Illustration | ‘‘Sometimes you can’t believe what happens and the only thing you can do is laugh.’’ (p391) |
| Finding (42) | Coped by for example “getting good news”, “denial”, “choosing no to think about it and “choosing what to hear” [C] |
| Illustration | “getting good news”, “denial”, “choosing no to think about it and “choosing what to hear” (p393) |
| Finding (43) | Participants also compared their experience with other patients and caregivers with advanced pancreatic cancer, particularly those ‘‘who were sicker or who died.’’ This engendered a ‘‘sense of gratitude’’ that ‘‘things could be worse.’’ [C] |
| Illustration | “who were sicker or who died.’’ This engendered a ‘‘sense of gratitude’’ that ‘‘things could be worse.’’ (p393) |
| Finding (44) | What was important to all participants was “living in the present” and “looking for the good in every situation.” [U] |
| Illustration | “living in the present” and “looking for the good in every situation.” (p393) |
| Finding (45) | Speaking to ‘‘positive people’’ gave them strength and offered a supportive perspective. [U] |
| Illustration | People were ‘‘more supportive than could ever be imagined.’’ (p391) |
| Finding (46) | the idea of coping was not an active process but something ‘‘you just have to do what other choice do you have.’’ [U] |
| Illustration | ‘‘you just have to do what other choice do you have.’’ (p391) |
| Finding (47) | coping involved stifling emotions and maintaining their composure, particularly in the presence of the patient. |
| Illustration | No illustration (unsupported) |
| Finding (48) | three of the participants were not coping well. Their distress was apparent in their words and voices. (unsupported) |
| Illustration | No illustration (unsupported) |
| Finding (49) | participants discovered their own strength, experienced the support of others, appreciated time together, were strengthened by the patients themselves, and had a different perspective on life. [C] |
| Illustration | ‘‘In the face of death, you learn to not sweat the small stuff’’ (p391) |
| Finding (50) | Participants captured a central idea of ‘‘just appreciating being together’’ for whatever timewas left. [U] |
| Illustration | A wife stated ‘‘I have never spent this amount of time with him and I am grateful because we have actually become closer.’’ (p393) |
| Finding (51) | Discoveries, gains, or growth were secondary outcomes which could only be appreciated based on prior lived experiences and losses. |
| Illustration | No illustration (unsupported) |
| Finding (52) | When asked about the gains in caregiving, the overwhelming response by family caregivers was that there were no gains. |
| Illustration | No illustration (unsupported) |
| Finding (53) | Personal growth was expressed as caregivers experienced new perspectives about life. [C] |
| Illustration | ‘‘why me’’ as her mother said ‘‘no, why not me?’’ (p394) |
| Finding (54) | Transitions were, for the most part, not specific events but more gradual changes over time |
| Illustration | No illustration (unsupported) |
| Finding (55) | The greatest transition was when it was clear that the patient’s symptoms resulted from the disease rather than the treatment. [C] |
| Illustration | ‘‘cancer showing on the outside,’’ (p394) |
| Finding (56) | a shift of focus from society, the community, and family to a laser-like focus on the patient and relationship. [C] |
| Illustration | ‘‘every ounce of energy into sustaining the relationship.’’ (p394) |
| Finding (57) | caregivers had fleeting thoughts about the patient’s death, and what this would mean in terms of their own identity and life. |
| Illustration | No illustration (unsupported) |
| Finding (58) | a major transition was coming to a point of acceptance that death was near and patients were ready themselves to ‘‘let go.’’ [U] |
| Illustration | ‘‘She is telling me what she wants and as hard as it is to hear, it helps me understand what I need to do.’’ (p391) |
| Finding (59) | Caregivers could not readily identify their own unmet needs |
| Illustration | No illustration (unsupported) |
| Finding (60) | caregivers’ unmet needs were to have someone to address their feelings and acknowledge the physical, emotional, social, and spiritual impacts associated with illness, loss, and the anticipated death of someone they loved. |
| Illustration | No illustration (unsupported) |
| Finding (61) | all participants welcomed the opportunity to share their true feelings, one going so far as to say that participating in the study ‘‘was a blessing.’’ [U] |
| Illustration | Participating in the study ‘‘was a blessing.’’ (p394) |
| **Article Reference:** | **(J) Shih *et al* (2013)** |
| Finding (1) | There is large impact on family members when their beloved one recently diagnosed advanced terminal stage HCC. |
| Illustration | No illustration (unsupported) |
| Finding (2) | patient’s signs and symptoms of HCC, disease exacerbated and treatment delayed. Therefore, participants felt guilty and blamed themselves. [U] |
| Illustration | “He went climbing this June, traveled abroad in July, and did push-up regularly. I don’t believe he got liver cancer not even on terminal stage. I know he always likes to drink alcohol secretly. It’s my fault not prohibiting him from drinking. I should have removed all bottles of alcohol from home.” (p4657) |
| Finding (3) | felt their lives were seriously disrupted by their loved one’s illnesses, which resulted in increased responsibilities, rearranged priorities, and an inability to plan. [U] |
| Illustration | “It’s changed my daily routine. It totally disrupted my life. I have to rearrange a lot of things such as my kids, my work, and getting help for my house cleaning … doing all the grocery shopping. I have to make sure my kids’ routine life such as their homework, going to and coming back from cramp school and band lesson as regular as possible.” (p4657) |
| Finding (4) | they shifted their priorities to adapt this shock diagnosis [U] |
| Illustration | “I need to take days off to take care of my father and I ask my wife to cook favorite dish for my father. I ask my daughter to be more independent because I don’t have much time to take care of her. I ask my subordinate to take more responsibilities for my job.” (p4657) |
| Finding (5) | difficulty in planning ahead since cancer is a severe disease to affect their lives [U] |
| Illustration | “I’m afraid to make vacation or take business trip. I am scared of anything happen to him during my absence. I just don’t have plan now.” (p4657) |
| Finding (6) | When doctor announced patient’s diagnosis, possible treatments, and prognosis, most family members were shocked and could not accept the news |
| Illustration | No illustration (unsupported) |
| Finding (7) | participants wanted to search all possible regimens, such as Chinese herbs,healthy supplements, certain foods, religion, etc. [U] |
| Illustration | “Doctor said to me that my father is in acute stage. His condition needs to be controlled first before getting TACE therapy. So, I went to temple and asked for my favorite goddess which Chinese regimen is best for my dad and what he can eat or not to eat. As long as it works, I would try my best to buy it for him.” (p4657) |
| Finding (8) | A number of family members indicated their feelings of helplessness and yet they tried to search all kinds of possible regimens. [U] |
| Illustration | “I feel helpless. I don’t know how to help him.” “I heard Antrodia cinnamomea essence extract is good for liver cancer and I also did the research and made sure it’s true. I’ve already bought one box for him.” (p4657) |
| Finding (9) | when doctor announced patient’s diagnosis as newly diagnosis advanced terminal stage hepatocellular cancer, they felt unbearable and were filled with dismay. [U] |
| Illustration | “He still has a lot of plans waiting for him to be completed and I want to accompany him to travel. I never thought his condition was so bad. I hope there is still chance for him.” (p4658) |

| **Article Reference:** | **(K) Larsen *et al* (2020)** |
| --- | --- |
| Finding (1) | Throughout illness and treatment, relatives lived with anxiety and fear of losing the patient.[C] |
| Illustration | The worries were terrible. I went for walks for hours, sobbing uncontrollably, (p4) |
| Finding (2) | They had concerns about the future and were more affected emotionally than the patient. [U] |
| Illustration | Well, my husband has been cool and collected. I think I was more affected. (p4) |
| Finding (3) | Relatives were burdened by anxiety and uncertainty regarding the future |
| Illustration | [Unsupported] |
| Finding (4) | [Relatives] were often isolated with their anxiety, always in consideration for the patient. [U] |
| Illustration | I talked to the children and my husband about it, but my husband couldn’t understand my sorrow. (p4) |
| Finding (5) | Relatives were confronted not only with the mortality of the patient but also with their mortality. [U] |
| Illustration | The possibility is there for one of us dying quickly (p4) |
| Finding (6) | This caused reflections about economy and practical issues [U] |
| Illustration | We talked about if we should stay on at the house or sell it. (p4) |
| Finding (7) | Confronting and talking about the worst-case scenario helped relatives deal with EC and treatment. [U] |
| Illustration | I think it’s a good thing to have discussed, even if it isn’t pleasant. (p4) |
| Finding (8) | Some relatives expressed their sorrow and possible loss of togetherness [C] |
| Illustration | Well, I have thoughts about how long we can walk side by side. This has been on my mind. (p4) |
| Finding (9) | Relatives were always alert and trying to adjust when something unexpected happened. [C] |
| Illustration | The thoughts are about what will happen. Can I keep him alive? (p4) |
| Finding (10) | Sometimes patients with EC fought single-handedly with their fears [C] |
| Illustration | The worries were terrible. I went for walks for hours, sobbing uncontrollably…(p4) |
| Finding (11) | When patients withdraw from the family, relatives were precluded from sharing their fear leading to feelings of isolation and loneliness. [C] |
| Illustration | He isolated himself during the treatment. He had been pre-occupied when we talked to him, not present at all. (p4) |
| Finding (12) | Relatives experienced a loss of togetherness, leading to an even more burden-some period. |
| Illustration | Unsupported |
| Finding (13) | Relatives acknowledged the necessity of considerations about possible death. [U] |
| Illustration | You have to talk within the family about the possibility of a poor outcome. (p4) |
| Finding (14) | Facing the uttermost fear helped relatives confront illness and loss of certainty about the future. |
| Illustration | Unsupported |
| Finding (15) | Relatives and patients shared the burden of the pervasive awareness of the illness and treatment course, and they could face the fear of a possible lethal outcome together |
| Illustration | Unsupported |
| Finding (16) | During the patients’ treatment with chemotherapy, relatives were preoccupied with time schedules for medication and treatment. |
| Illustration | Unsupported |
| Finding (17) | They saw the treatment schedule as a joint event, supporting the patient to remember medication at the appointed hours. [U] |
| Illustration | We knew it was vital for him to eat, so we looked at the clock all the time. (p5) |
| Finding (18) | [scheduling around medication and appointments] could be experienced as a strain on and disruption of everyday life [C] |
| Illustration | It was like being slaves of the clock. (p5) |
| Finding (19) | Because of the treatment schedule, relatives experienced a restricted daily life, adjusting to new routines [U] |
| Illustration | It was like being slaves of the clock. (p5) |
| Finding (20) | Relatives were on the sidelines, undertaking responsibility for treatment to a large degree |
| Illustration | Unsupported |
| Finding (21) | Especially the burdensome task of eating was perceived as a mutual struggle [U] |
| Illustration | He cannot figure out about sizes and frequency of meals... We find it difficult to figure out what he can eat. (p5) |
| Finding (22) | Troubles with eating became a joint concern, and relatives were deeply engaged with adjusting to new eating habits [U] |
| Illustration | We knew it was vital for him to eat, so we looked at the clock all the time. (p5) |
| Finding (23) | [Relatives are] undertaking a huge responsibility, and constantly alert to whether the patients lost or gained weight. [U] |
| Illustration | The food thing is omnipresent. We have been told that he is not allowed to have further weight loss. (p5) |
| Finding (24) | The worry about patients’ weight was, largely, associated with patients’ progress and recovery, making meals into events burdened with anxiety and despair. [U] |
| Illustration | I am watching what he eats, constantly pushing him... It’s frustrating that he has lost so much weight. (p5) |
| Finding (25) | If the patient was struggling, that could destroy hope for progress [C] |
| Illustration | I had a hard time with the food and meals, sitting there, seeing he couldn’t get the food down in his throat (p5) |
| Finding (26) | Some relatives could not bear to see the patient’s struggle [U] |
| Illustration | I couldn’t stand looking at him battling with the food. I have been deeply emotionally affected by this. (p5) |
| Finding (27) | The former relationship between relatives and patients could change. The changed life situation caused relatives to reflect on the distribution of roles within the family. [U] |
| Illustration | My husband has always been in charge of the expenses; he really likes that. I used to be okay with him in charge. But after he got sick, I demanded that he introduced me to the financial part… (p5) |
| Finding (28) | Claiming control seemed to be part of coping with a changed life situation. Knowing that they could manage areas such as expenses made them more at ease with the threat of cancer. [C] |
| Illustration | I have become the person who looks after him. In a way, I’ve become his relief organization, so his life can move on as smoothly as possible. (p5) |
| Finding (29) | A changed balance within the family, and sometimes wives thought they took a motherly role towards their patient husband. [U] |
| Illustration | Sometimes I felt like a mother talking to a child: “Remember to do this and that.” (p5) |
| Finding (30) | The position as buffer entailed the relative’s serving as a target for patients’ frustrations, trying to be supportive and optimistic. [U] |
| Illustration | I try to calm him down, and sometimes he calls me a stupid cow. Then, I try to talk with him to make him more at ease with the situation. (p5) |
| Finding (31) | Relatives were constantly in a position of hiding their fears and anxiety, wanting to be strong and authoritative, always believing in treatment |
| Illustration | Unsupported |
| Finding (32) | Relatives did not think they should be active in the hospital context. [U] |
| Illustration | I try to be in the background during consultations. (p6) |
| Finding (33) | They took a passive position, not asking questions, awaiting the directives of health professionals. [C] |
| Illustration | He has the illness. He decides and has the authority. It’s his body under the influence of treatment. (p6) |
| Finding (34) | Some relatives expressed a lack of courage to pose questions [U] |
| Illustration | perhaps we lacked the courage to ask. (p6) |
| Finding (35) | Relatives positioned themselves on the sideline, subjected to the authority of both the healthcare professional and the patient. [U] |
| Illustration | He has the illness. He decides and has the authority. It’s his body under the influence of treatment (p6) |
| Finding (36) | They depended on patients and healthcare professionals to give them space and acknowledge their roles as relatives. [U] |
| Illustration | If you got up, then the health professionals would say hello. They didn’t invite you, nor were they dismissive of you. But if you are quiet and don’t follow the patient, then I don’t know if they know you are on the side-line. They don’t ask. (p6) |
| Finding (37) | Some relatives stated that they needed to call attention to themselves. [U] |
| Illustration | But if you are quiet and don’t follow the patient, then I don’t know if they know you are on the side-line. (p6) |
| Finding (38) | Relatives did not feel rejected, but they reflected on the missing invitation to be part of the treatment course and decision-making. [C] |
| Illustration | No health professionals involved me in this decision. (p6) |
| Finding (39) | If relatives were to participate, they needed to authorise themselves, making themselves visible and claiming their roles as relatives. [U] |
| Illustration | my husband could ask questions, but I didn’t have the space to ask questions, not unless my husband allowed it. (p6) |
| Finding (40) | The missing empowerment by patients and health professionals caused relatives to have unanswered questions. [U] |
| Illustration | my husband could ask questions, but I didn’t have the space to ask (p6) |
| **Article Reference:** | **(L) Wong *et al* (2020)** |
| Finding (1) | Caregivers feared losing their loved ones [U] |
| Illustration | the fear of not being sure of how it’s going to happen and how I’m going to react...I’m afraid of losing him (p5) |
| Finding (2) | Caregivers watched from the side-lines as they too felt helpless in their inability to control treatment or disease side effects [U] |
| Illustration | Well, I am helpless. I can give her emotional support but I can’t cure the cancer, I can’t make her not nauseous, I can’t make her go to the bathroom... (p5) |
| Finding (3) | The fixed schedule and inability to travel produced a sense of confinement as they highlighted needs to escape from current reality. |
| Illustration | Unsupported |
| Finding (4) | Balancing relationships was difficult as caregivers tried to maintain their “husband-wife relationship” without it becoming too much of a “patient-nurse relationship” |
| Illustration | Unsupported |
| Finding (5) | The sudden adjustment to learning new medical information ... [U] |
| Illustration | “mentally draining and extremely frustrating” (Jason) (p6) |
| Finding (6) | caregivers felt like they were one person trying to assume five different roles of a nurse, secretary, and pharmacist, all while maintaining who they were and picking up the patient’s previous tasks. [C] |
| Illustration | Ian presented a photograph of a deconstructed motor to illustrate caregiver burnout (Fig. 3): “Where do we begin? We need help!” (p6) |
| Finding (7) | Although caregivers recognized the need for self-care, they tended to “put ourselves on the back-burner” (Helen) [U] |
| Illustration | they tended to “put ourselves on the back-burner” (Helen) (p6) |
| Finding (8) | lack of [direct] interactions with medical providers [C] |
| Illustration | Even when you go in and see Dr. X or any of the other providers, it’s about them [the patients]. Nobody says how are you doing?” (p7) |
| Finding (9) | Although caregivers reported having good social support from family and friends, they appreciated being in a caregiver group because they didn’t want to burden their family and felt like others wouldn’t understand. |
| Illustration | Unsupported |
| Finding (10) | Caregivers reported becoming more vigilant of new symptoms, aches, or pain as treatment progressed |
| Illustration | Unsupported |
| Finding (11) | Although they were excited that the patient was doing well, they were also skeptical of the situation, even when presented with good news from the medical team. [U ] |
| Illustration | Jacklyn’s photograph of a dark cloud (Fig. 4) to signify the constant overshadow of doubt and worry - No matter what kind of wonderful day you’re having, you know that these black clouds are there and on any day, life could change again in a minute. So you never ever really are without feeling that. (p7) |
| **Article Reference:** | **(M) Winterling *et al* (2004)** |
| Finding (1) | Many patients and spouses were aware of the poor prognosis but some seemed to believe that there was a chance of a cure [U] |
| Illustration | One would like to do more things together when he gets well, so we have already started to look for concerts and stuff in springtime. (p384) |
| Finding (2) | They also said that there was a risk that the patients might die of the illness. |
| Illustration | Unsupported |
| Finding (3) | A few spouses stated that they thought that their partners would be cured. [U] |
| Illustration | One would like to do more things together when he gets  well, so we have already started to look for concerts and  stuff in springtime. [Spouse (19) 10] (p384) |
| Finding (4) | Some of the spouses stated that they suffered from tiredness |
| Illustration | Unsupported |
| Finding (5) | Some spouses that they had lost interest in everything, except their sick partner. |
| Illustration | Unsupported |
| Finding (6) | Many spouses stated that they suffered when they saw the patients suffer from fatigue, pain, etc. [U] |
| Illustration | It’s very sad to see one’s partner so sick, she gets nausea when she sees food and can’t eat properly, it’s terrible (p384) |
| Finding (7) | Some spouses said that they wanted to be able to do more for the patients and some wondered if they did enough. |
| Illustration | Unsupported |
| Finding (8) | Informants felt that this was unfair since they already had another serious illness that influenced their life, that there were other people who were older and healthier, or felt disappointed at not being able to do things they planned for [C] |
| Illustration | Why does this happen right now ... now when life is at its best. (p384) |
| Finding (9) | A few said that God had given them a trial that was too hard. |
| Illustration | Unsupported |
| Finding (10) | Another wondered if the illness was some sort of punishment. [C] |
| Illustration | Sometimes I wonder where the cancer came from and if I have done something wrong … but it’s not possible to say. Anybody can get it whether one is smoking or  not, eating badly or not. Anyone can get it apparently.  (P 10) (p384) |
| Finding (11) | A few informants said that they had not at all thought about why, and others that there was no use thinking about it since there is no answer |
| Illustration | Unsupported |
| Finding (12) | Brooding about “why” also included wondering about the origin of the cancer. [U] |
| Illustration | Sometimes I wonder where the cancer came from and if I have done something wrong ... but it’s not possible to say. (p384) |
| Finding (13) | Both patients and spouses wondered about how the illness would develop, for how long the patients would live, and what the disease would do to the patients physically. |
| Illustration | Unsupported |
| Finding (14) | The spouses stated that they did not want to be left alone in life and they wondered if they would be capable of organizing all the practical things. [U] |
| Illustration | I won’t manage I thought at first, then I thought yes of course I will manage, but how will life go on? ... Then I thought of all of those who have gone through it ... they have made it, but I must leave all that I love so much, the house and the farm after my partner dies. (p385) |
| Finding (15) | spouses had to organize and carry out almost all the practical things that needed to be done... The spouses said that they wanted to help the patient because it was their duty as a partner. [U] |
| Illustration | I don’t do it in order that he should be grateful, I do it because I should do it, I want to do it and I will, I must have the strength and I have said that I simply must have the strength. (p385) |
| Finding (16) | They also felt that they had to manage although they were tired and worn out themselves. |
| Illustration | Unsupported |
| Finding (17) | They found it bothersome because they did not feel that they wanted to help that much, they felt locked in or so much in despair themselves that they could not carry on anymore. |
| Illustration | Unsupported |
| Finding (18) | their marital relationship had improved or that they finally got peace in their life. [U] |
| Illustration | we are just as close as before ... well, even closer (p385) |
| Finding (19) | One just has to accept it or that the illness was part of the suffering and misfortune of normal life that one has to reckon with. [U] |
| Illustration | Every human has to go through it sometimes. (p385) |
| Finding (20) | They wanted to accept the situation but felt that they could not. [C] |
| Illustration | In peace and quiet, yes, it has to be as it is right now, it’s ok but it can change, one does not know anything ahead. (p385) |
| Finding (21) | Few expressed a kind of confidence that everything would come out all right. [C] |
| Illustration | I don’t think I’m religious but I have faith because I believe in some way that there is some power that is with you (p385) |
| Finding (22) | One should not give up, but look ahead, be strong, and have a positive outlook. [U] |
| Illustration | We live day by day now. Talk a little and ... try to make the best of the situation. (p385) |
| Finding (23) | Some said that they tried to keep their spirit up. [C] |
| Illustration | We live day by day now. Talk a little and ... try to make the best of the situation. (p385) |
| Finding (24) | They tried to take care of themselves, take some time of their own, so that they had the strength to help the patient |
| Illustration | Unsupported |
| Finding (25) | the couple should live together as long as possible and be able to do things together. |
| Illustration | Unsupported |
| Finding (26) | A few expressed the hope that the patient would be cured. [U] |
| Illustration | That they can perhaps put a stop to it (the illness) for a while ... so that is what one hopes for and above all that she will be a little better and have a better life than now. (p386) |
| Finding (27) | The informants had planned for where and how the patients should be buried, they had made a will, and sorted out the economy. |
| Illustration | Unsupported |
| Finding (28) | A few spouses stated that this was not possible because the patients had not understood the prognosis. |
| Illustration | Unsupported |
| Finding (29) | They sought support from talking to their partners, the rest of the family, friends, or healthcare staff |
| Illustration | Unsupported |
| Finding (30) | A few prayed to God for consolation and cure. |
| Illustration | Unsupported |
| Finding (31) | Some relatives sought support by having other persons as company. |
| Illustration | Unsupported |
| Finding (32) | ….spouses mentioned that they received support from their family, friends, and the healthcare staff, both psychologically and practically. |
| Illustration | Unsupported |
| Finding (33) | The informants tried to avoid thinking about the illness and did things to distract themselves. [U] |
| Illustration | Unsupported |
| Finding (34) | The informants tried to carry their burden all alone. [U] |
| Illustration | Whatever they tell me, only I can handle this, there is nobody who can help me, no there isn’t and that is tough to realize. (p386) |
| **Article Reference:** | **(N) Nolan *et al* (2006)** |
| Finding (1) | Promises of prayer and requests for prayer were some of the most common spiritual issues observed in the postings [U] |
| Illustration | One poster said that she was desperate when she asked for prayers for her loved one, whom she described as declining rapidly (p241) |
| Finding (2) | A number of posters acknowledged that their fellow chat room contributors may have diverse spiritual and religious practices [U] |
| Illustration | by prefacing their request for prayers for a loved one with statements such as, “For those of you who pray.” (p241) |
| Finding (3) | Several posters shared with others the blessings for which they were thankful [C] |
| Illustration | such as a supportive friend, a helpful nurse, or being able to take time off work to be with their loved ones (p241) |
| Finding (4) | Some postings described feelings of conflict between spiritual beliefs and the present experience of suffering but seemed to present the conflict to other posters in an open-minded or hopeful way. [C] |
| Illustration | one poster stated that she had reached her limit for tolerating stress but believed that God did not give a person more suffering than he or she could handle. (p242) |
| Finding (5) | Two posters questioned why God wanted their  loved ones to suffer. [C] |
| Illustration | One expressed concern that God was  punishing her mother with this disease. Still another described  his father’s life as one in which he gave of himself to many  of those around him but received little thanks in return. (p242) |
| Finding (6) | Two posters personified pancreatic cancer [U] |
| Illustration | as “the monster,”; one asking God why her loved one had to fight that monster. (p242) |
| Finding (7) | Two others also asked God to take their loved ones [C] |
| Illustration | Two others also asked God to take their loved ones because they did not want to see them suffer any longer. (p242) |
| Finding (8) | Several posters cast the suffering of their loved ones in a more positive way [C] |
| Illustration | explaining that they had gotten to know their loved ones more intimately by helping them through the illness experience to death (p242) |
| Finding (9) | For some, hope seemed all encompassing [U] |
| Illustration | e.g., trying to have new hope each day; Several posters simply closed their message with statements such as “Keep the faith,” or “There is hope out there.” (p242) |
| Finding (10) | expressed more specific hopes [C] |
| Illustration | that new treatments would be found to cure their loved ones before death. (p242) |
| Finding (11) | their loved ones were dying but hoped that their pain could be controlled during their last months of life [C] |
| Illustration | One poster stated that her mother hoped that she could be pain-free but remain alert enough to interact with her family. (p242) |
| Finding (12) | the concept of “false hope.” [C] |
| Illustration | The poster was the friend of an older adult with pancreatic cancer whom she thought was receiving aggressive chemotherapy that served only to prolong her suffering. She stated that she thought that having hope was important but that her friend’s physician was giving her false hope. (p242) |
| Finding (13) | the acceptance of pancreatic cancer or impending death as being the will of God. [C] |
| Illustration | One poster explained that her husband had complete trust in God and sought to follow God’s will even as he suffered from his illness. (p242) |
| Finding (14) | postings reported the death of a loved one with pancreatic cancer. [U] |
| Illustration | One poster reported her father’s day of death as the day he was “born into eternal life.” Another described her loved one’s transition as a passing into the next world with the help of angels (p242) |
| **Article Reference:** | **(O) Morowatisharifabad *et al* (2020)** |
| Finding (1) | The role of individual performance in incidence of disease [U] |
| Illustration | “I think that addiction is the most important reason of my dad’s disease. Most of the time, he is engaged in drug addiction and he takes large doses of drugs. (p3) |
| Finding (2) | The role of hereditary factors in incidence of disease [U] |
| Illustration | I think genetic factors can be effective because I heard from my father that my grand-father had stomach cancer, and my aunt had lung cancer. My grandparents have a family relationship, and some of our relatives died of cancer. (p3) |
| Finding (3) | Prevalence of the disease due to living area [U] |
| Illustration | I think because the climate of our province is humid, microbial growth is greater here, but in cities that are sunny or dry, microbes are destroyed... (p4) |
| Finding (4) | people stated that our province has more rural areas because of the good climate and soil fertility conditions and people are often busy with many activities. [U] |
| Illustration | In rural areas, people are very active and they work from morning till evening, they sometimes do not care for their nutritional needs or food health, so the incidence of these diseases are higher there. (p4) |
| Finding (5) | Tensions … of life. [U] |
| Illustration | The mother of all diseases is stress while everyone is suffering from it. My mother was very stressed out and even she was concerned about the repayment of loans we had taken for her disease treatment... (p4) |
| Finding (6) | ….pressures of life. [U] |
| Illustration | “I think that troubles in life are the most important issue because having a child with a severe disability make us tired of living... ... Of course financial issues are also important, because if you do not have a financial problem, you can overcome some of the problems. For example, I can hire a babysitter to care my child …”[P16]. (p4) |
| Finding (7) | Lack of nutritional self-care [C] |
| Illustration | “My husband drinks his tea hot but now, after his disease,  he tries to break this habit…”[P5]. (p4) |
| Finding (8) | Even participants complained about that food products that are available in the market [U] |
| Illustration | we consume oil a lot in our foods, especially solid oil, but we rarely eat vegetables and fruits, less than once a week... (p4) |
| Finding (9) | Low monitoring of food available in the market [U] |
| Illustration | I think there is not enough monitoring over food products available in the market by the government...there are poisonous foods or hormones in meat products, and even human and human fertilizers are used in products... (p4) |
| Finding (10) | Participants described disease-related problems in their patients, including physical problems and apparent changes due to illness [U] |
| Illustration | After each chemotherapy session, my father cannot eat at all for about a week, the only thing he eats is a little juice. The enlargement of the tumor makes him unable to eat something. He sometimes feels fatigue, and he lost a lot of his weights. (p4) |
| Finding (11) | The emergence of other diseases during chemotherapy [U] |
| Illustration | after treatment, she cannot eat at all. She has severe diarrhea and vomiting that are related to her treatment. (p4) |
| Finding (12) | Metastasis of the disease to other parts of the body [U] |
| Illustration | Every time that we perform a CT scan, we understand that the disease condition gets worse and now her lungs are involved (p4) |
| Finding (13) | Psychological problems after being the disease [U] |
| Illustration | Her doctor said that she should have patience and hope to overcome the disease. (p4) |
| Finding (14) | Need high spirits to endure disease [U] |
| Illustration | My mother, after a few sessions, got tired and did not want to continue the treatment. She is afraid of not getting well, but she continues her treatment because of my insistence. (p4) |
| Finding (15) | Hate life due to pain and discomfort [U] |
| Illustration | Her mood is good enough now, but on the first days after being diagnosed with cancer, she wanted God to die to not experience these pains (p4) |
| Finding (16) | Feeling hopeless and considering the disease as untreatable. [U] |
| Illustration | Doctor did not hope that our patient would be alive, but said that she would have less eating problems after treatments (p4) |
| Finding (17) | Enduring great difficulty during treatment. [C] |
| Illustration | Commuting every day from our village to the chemotherapy center is very difficult for us, because our village is far from the treatment center. (p4) |
| Finding (18) | Long-term treatment of the disease [U] |
| Illustration | This kind of diseases need continuous follow-up, frequent commuting, and long waiting for physician’s visit (p4) |
| Finding (19) | Persistent medical check-up [U] |
| Illustration | CT scan should be done every few months. My mother is bored and tired of frequent check-ups and examinations. (p4) |
| Finding (20) | Conducting surgery and removing tumor [U] |
| Illustration | After performing experiments, the doctor said that the disease had progressed and there was a need for surgery ... My mother immediately underwent surgery (p4) |
| Finding (21) | Cancer as a life disturbance [U] |
| Illustration | “You must experience this illness to understand how  agonizing it is. Except for the discomfort caused by the disease,  now our whole lives are involved with the illness of my  mother, because we all see her sufferings ...”[P15]. (p4) |
| Finding (22) | Participants acknowledged that patients regarded themselves as being a burden on their families. [U] |
| Illustration | My mother does not like being a burden on her family so she does not go to doctor unless she cannot tolerate her pain. |
| **Article Reference:** | **(P) Gooden *et al* (2013)** |
| Finding (1) | Managing diet, difficulties around eating and gastrointestinal discomfort impacting on quality of life emerged as the most prominent themes in both groups. |
| Illustration | Unsupported |
| Finding (2) | Participants talked about experiencing symptoms of fat malabsorption and steatorrhoea [U] |
| Illustration | ...she couldn’t eat anything with fat in it, so she was eating very bland food. Her faeces were extremely smelly. (p1837) |
| Finding (3) | Participants described struggling with symptoms of malabsorption unaware that they could be relieved or helped with medication |
| Illustration | Unsupported |
| Finding (4) | Did utilise the internet to source information about diet and gastrointestinal symptoms and it was online that they first found out about pancreatic exocrine insufficiency (PEI) [U] |
| Illustration | “They (family) went on the internet and searched and  they rang up a couple of people and that’s when they  found out about the pancreatic enzymes.” Carer, Male  (Bereaved) (p1838) |
| Finding (5) | Problems with diet were compounded by a lack of access to dieticians or routine assessment in the clinical setting and/or follow-up post-discharge [U] |
| Illustration | I think there was one dietician or two dieticians or something in the whole hospital. You know the lack of staff, this is the story. (p1838) |
| Finding (6) | Participants expressed frustration that dietary advice and assessment were not forthcoming. |
| Illustration | Unsupported |
| Finding (7) | They also expressed anger that they were not given basic information about the effects of pancreatic exocrine dysfunction (or treatment) that could have relieved unnecessary suffering and discomfort and improved their quality of life. |
| Illustration | Unsupported |
| Finding (8) | Participants described symptoms of PEI as causing them discomfort or distress. |
| Illustration | Unsupported |
| Finding (9) | This distress was exacerbated by a perceived reluctance of clinicians to prescribe EST and the lack of understanding as to the need for management of PEI. [U] |
| Illustration | We brought up with him ‘pancreatic enzymes’. He [clinician] was- Oh I don’t know whether that’s a good idea. We explained a few things and he seemed very reluctant to give them. (p1838) |
| Finding (10) | Participants described how persistent and proactive they needed to be in accessing information and advice and how they had to ‘keep asking’ or ‘go elsewhere’ to access effective management for their symptoms. [U] |
| Illustration | We went back to the oncologist and we just put it on her about the pancreatic enzymes, never mentioned that we had mentioned to the palliative care doctor about it and she was quite happy to write a script out for it and that was a very good benefit. (p1838) |
| Finding (11) | There were inconsistent levels of understanding regarding their [pancreatic enzyme substitution therapy] appropriate and effective use |
| Illustration | Unsupported |
| Finding (12) | Properly supervised EST did show stabilisation of or increase in weight, resolution of pain, diarrhoea and abdominal discomfort [U] |
| Illustration | The Creon®, we found helps. She hasn’t lost a lot of weight.  Well the pancreatic enzyme [Creon®] made such a huge difference. If we’d only known we could have got that earlier. That would have been great. (p1838) |
| Finding (13) | For the carer/family group, concerns around managing diet and symptoms of PEI were linked to increased carer burden and significant distress. Participants expressed feelings of anger, frustration and powerlessness, as they struggled to find foods for their loved ones to eat that minimised symptoms. [C] [U] |
| Illustration | “I found this very confronting, her not wanting to eat. I  (crying) try to force feed her. She gets upset, it makes  it worse.” Carer, Male (Dyad) (p1839) |
| Finding (14) | It was also flagged as causing the most tension and difficulties in relationships between carer and patient in the dyad subgroup [U] |
| Illustration | I found this very confronting, her not wanting to eat. I(crying) try to force feed her. She gets upset, it makes it worse. (p1839) |
| Finding (15) | Participants’ lack of knowledge about the effects of pancreatic disease on eating and digestion meant some of them continued struggling with symptoms, in ignorance [U] |
| Illustration | We only got it [pancreatic enzyme supplement] about a week or 10 days out [before she died] and it would have been better if we’d had them a month earlier. (p1839) |
| Finding (16) | For the bereaved group, it was this particular issue that stood out for them as having increased their distress contributing to their feelings of unresolved grief. [C] |
| Illustration | We only got it [pancreatic enzyme supplement] about a week or 10 days out [before she died] and it would have been better if we’d had them a month earlier. (p1839) |
| **Article Reference:** | **(Q) Hodgson (2006)** |
| Finding (1) | They identified how life revolved around food [C] |
| Illustration | ‘...constant attention to healthy nutrition.’ (p1159) |
| Finding (2) | The need for constructive advice was important and the specialist nurses were much appreciated along with the support from the OPA. |
| Illustration | Unsupported |
| Finding (3) | Accepting that recovery is slow, and learning to sleep on a raised bed takes time, but the support from other carers and specialist nurses is most valued. |
| Illustration | Unsupported |
| **Article Reference:** | **(R) Hansen *et al* (2017)** |
| Finding (1) | Spousal caregivers most often described their relationship as emotionally close and supportive. |
| Illustration | Unsupported |
| Finding (2) | Most non-spousal caregivers described having an emotionally distant, non-supportive relationship with the patient. [C] |
| Illustration | I’d be changing his pants six and seven times a day, and [the patient] would just complain about it all the time, about how he [had another bowel movement in] his pants again. So, I don’t know. Maybe he’s getting his karma if you want to look at it that way. (p565) |
| Finding (3) | Underscoring some relationships was the patients’ history of substance abuse and family conflict. [C] |
| Illustration | So, I don’t know. Maybe he’s getting his karma if you want to look at it that way. (p565) |
| Finding (4) | For those with an emotionally distant relationship, tension between the patient’s history and the caregiver’s struggle to provide care frequently surfaced. |
| Illustration | Unsupported |
| Finding (5) | All family caregivers reported having little knowledge of HCC and little to no knowledge of providing care to people with terminal HCC [U] |
| Illustration | My concern at the beginning was— [the patient had] lived here for three months, slept on the couch [while] we knew nothing about [HCC]. I did do some research on the computer and learned how serious it was. (p565) |
| Finding (6) | Many described receiving little information from HCPs and seeking out information from alternative sources (e.g., the Internet, books) to learn about HCC, symptoms, and treatments. [U] |
| Illustration | I did do some research on the computer and learned how serious it was. (p565) |
| Finding (7) | A few family caregivers mentioned the stigma of an HCC diagnosis and substance abuse. [U] |
| Illustration | You know, when you say cirrhosis of the liver, they think, ‘Oh, you drank yourself . . .’ (p565) |
| Finding (8) | Felt compelled to inform friends and family that the patient’s diagnosis was not from alcohol abuse. [U] |
| Illustration | But we always had to make sure. We’d say that it wasn’t from drinking. It was because of fatty acids. You know, when you say cirrhosis of the liver, they think, ‘Oh, you drank yourself . . .’ (p565) |
| Finding (9) | A terminal HCC diagnosis brought a change in lifestyle, particularly as patients underwent treatments or required full-time care. [U] |
| Illustration | The thing that has been taken away from us in terms of lifestyle is the ability to be free. The ability to go somewhere when you want to go. . . . You can’t just do that anymore because [the patient] has a treatment program. . . . (p566) |
| Finding (10) | Some spousal caregivers stopped working to provide full-time care and had a difficult time adjusting to the change. [C] |
| Illustration | And it’s been difficult, but, at the same token, I know that I need to be home with him. And it’s been a rough time adjusting. (p566) |
| Finding (11) | Throughout the illness trajectory, family caregivers relied on visible physical changes as markers of how HCC was progressing but were frequently uncertain about how to identify and interpret symptoms. |
| Illustration | Unsupported |
| Finding (12) | Family caregivers identified few symptoms at the early stage of the disease. One non-spousal caregiver described the patient’s terminal HCC as invisible because the patient did not act sick or exhibit visible signs of illness. [C] |
| Illustration | I know it is liver cancer, it’s very deadly, and it’s hard to get through . . . but the way it affects my [relative], sometimes, I’m like, she doesn’t have cancer. I don’t really see it. (p566) |
| Finding (13) | As HCC progressed and patients’ symptoms worsened, family caregivers were uncertain about whether symptoms they could identify were related to the cancer. [U] |
| Illustration | It gets difficult to distinguish between what is diabetic-related and what is liver cancer–related, and, because he’s off his diet, that definitely affects how he feels. (p566) |
| Finding (14) | As HCC progressed to liver failure, family caregivers were uncertain whether symptoms were related to liver failure or substance abuse. [U] |
| Illustration | I don’t know if he’s getting encephalopathy or it’s just really the morphine. Because he said he takes the morphine, and then he has withdrawals and instantly he’s addicted, and he has the withdrawal symptoms for a few days. (p566) |
| Finding (15) | Without an understanding of HCC and its potential progression to liver failure, family caregivers had difficulty identifying what was happening and how to respond to unexpected complications. Hepatic encephalopathy was particularly challenging. [U] |
| Illustration | [The patient] was in and out of . . . what do you call it . . . encephalopathy, and doing some very weird things, like wandering out in the hallway to pee. We would just have to get up in the middle of the night and kind of take charge, and we did not know, really, anything about it. They didn’t have any literature. (p566) |
| Finding (16) | As the disease advanced and symptoms became more pronounced, family caregivers interpreted visible changes (e.g., sunken eyes, difficulty walking) as signs of worsening condition and markers of disease stage. [C] |
| Illustration | My child [was] doing research. He said one of the end things is the coma. . . . And then when [the hepatic coma] happened, I thought, ‘Well, geez, here we are to that stage now.’” (p566) |
| Finding (17) | Family caregivers frequently spoke about a lack of information from HCPs regarding treatments and their side effects. [U] |
| Illustration | And I don’t think that they really gave him very much information about how he might feel the next day, because he could just barely get out of his bed into the wheelchair. (p566/7) |
| Finding (18) | When patients’ condition worsened and TACEs were no longer effective, family caregivers contemplated the potential impact of chemotherapy and whether patients would be able to survive. [C] |
| Illustration | She is dizzy, doesn’t comprehend very well. I just don’t want the chemo to turn her into a sponge, and that’s just kind of the only thing that’s been on my mind. I hope she gets through it fine. (p567) |
| Finding (19) | Although most family caregivers were aware that the patient in their care would eventually die, some felt unprepared to provide end-of-life care themselves and continued to lack information about what to expect. [U] |
| Illustration | It was that scary to the point where I, I felt so helpless, and I just finally, I just say, ‘Maybe I need to take you into the hospital.’ I didn’t know what was going on.” (p567) |
| Finding (20) | As patients with a history of substance abuse neared end of life, pain management continued to be challenging and difficult for family caregivers to understand [U] |
| Illustration | I couldn’t understand why, I mean, when [the patient] had his procedures, they would give him fentanyl and this and that. Of course, I don’t want him to hurt. (p567) |
| Finding (21) | One non-spousal caregiver was surprised to learn that she would be responsible for providing such care. [U] |
| Illustration | I went, “You’re not supposed to call 911? What am I supposed to do? What if he just dies right here?” I mean, it seems they should have somebody say, “OK, if he’s with you, then here’s the procedure.” . . . [The nurse] gave me really no support about what to do. (p567) |
| **Article Reference:** | **(S) Gerhardt *et al* (2020)** |
| Finding (1) | During treatment and follow-up caregivers felt they were largely invisible to HCPs |
| Illustration | Unsupported |
| Finding (2) | they felt a tacit expectation from HCPs to undertake many practical and logistical tasks related to the patients' disease, treatment, care and rehabilitation whilst also having to manage housekeeping, work and support the patient psychologically. |
| Illustration | Unsupported |
| Finding (3) | HCPs seldomly approached caregivers proactively to inquire specifically about their needs. [C] |
| Illustration | It's not that they are unfriendly or anything, but they are just not there for caregivers (p3) |
| Finding (4) | HCPs were nevertheless always friendly and accommodating when caregivers themselves took the initiative to discuss or ask HCPs questions [C] |
| Illustration | It's not that they are unfriendly or anything, but they are just not there for caregivers (p3) |
| Finding (5) | Caregivers consequently felt they were silently enlisted as carers of the patients both during hospitalization and after hospital discharge. Their “job” included care of surgical drains, surgical wounds, nutritional and gastrointestinal function and postoperative pain management. [U] |
| Illustration | Well, you can't be on your own with that drain so I said: No, of course I'll take him home to my place......But what if something happens. What if he starts screaming with pain at two in the morning or if the drain falls out? What precisely do I do? (p3) |
| Finding (6) | They described doing so without adequate instruction and minimal interest from HCPs as to whether they performed the tasks correctly. [U] |
| Illustration | Well, you can't be on your own with that drain so I said: No, of course I'll take him home to my place......But what if something happens. What if he starts screaming with pain at two in the morning or if the drain falls out? What precisely do I do? (p3) |
| Finding (7) | Caregivers expressed a wish to also be given attention by the HCPs during follow-up. They articulated distinct needs for support including the opportunity to pose questions, discuss their concerns, get formal instructions regarding concrete technical care issues and information about potential future scenarios. Importantly, they suggested access to an open phone line to the hospital 24 h a day during follow-up would be helpful for caregivers. |
| Illustration | Unsupported |
| Finding (8) | Caregivers described that their worries and concerns were difficult for them to talk to anyone about. |
| Illustration | Unsupported |
| Finding (9) | During consultations in the healthcare system, caregivers experienced that HCPs avoided talking about the patient's prognosis, forcing caregivers to read between the lines. [U] |
| Illustration | “It was hinted that the surgery was really extensive – it wasn't just a  minor procedure. So, I caught on that this was serious” (p4) |
| Finding (10) | This influenced caregivers to refrain from asking questions about treatment options and prognosis although they as relatives felt a great need for this information |
| Illustration | Unsupported |
| Finding (11) | Being a caregiver and worrying about the future was stressful for caregivers, however they felt they should not burden the patients with their worries and concerns. |
| Illustration | Unsupported |
| Finding (12) | caregivers sought to gather information  about the disease from other sources, mainly from the internet.  They kept their findings to themselves without passing them on to the  patient. [U] |
| Illustration | “Sometimes I've searched the internet and discovered a lot of things. My  husband [the patient] would never do that … That's my way of finding  out what's going on” (p4) |
| Finding (13) | Some patients wished to attend follow-up visits on their own without the caregiver present. This made it more difficult for caregivers to keep tabs on the patient's situation and provide adequate support. [C] |
| Illustration | Some caregivers suggested that HCPs should explicitly encourage patients to bring caregivers to follow-up visits. (p4) |
| Finding (14) | Although caregivers themselves were anxious and fearful of disease recurrence, especially if patients developed worrying physical symptoms, they kept their anxiety and fears to themselves. [U] |
| Illustration | Through all of this I've only touched on my own feelings a few times and said I'm scared of losing you.......I know this isn't a “good” cancer....but I stay positive and say to him: Dad you're one of the good cases...They all said that you're a role model for other patients” (p4) |
| Finding (15) | Caregivers made it clear that their responsibility was to “stick with the patient”, be positive, put their own feelings aside and their lives on hold to care for and keep the patient's spirits up, what we call “keeping a stiff upper lip”. [U] |
| Illustration | “stick with the patient” “keeping a stiff upper lip”. (p4) |
| Finding (16) | This was energy-consuming for caregivers both physically and emotionally. Some experienced that they had difficulty sleeping and developed symptoms of stress and depression |
| Illustration | Unsupported |
| Finding (17) | Some re-sorted to alcohol to alleviate the stress, while others withdrew from socializing. [U] |
| Illustration | I drink more alcohol than I should...it's probably to get a bit of distance from it all when I think about it (p4) |
| Finding (18) | Caregivers reflected that, in hindsight, they potentially could have benefitted from talking to someone who was not personally involved in the disease trajectory, a professional of some sort. [C] |
| Illustration | a formalized “caregiver consultation” with a nurse or physician without the patient present might accommodate their need to discuss the challenges of being a relative. Specifically, they were convinced that a consultation at discharge addressing their role as caregivers, including where they as caregivers could seek help and support would be beneficial. (P4) |
