# Supplementary material for: Experiences of informal caregivers supporting individuals with upper gastrointestinal cancers: a systematic review
Source: BMC Health Serv Res. 2024 Aug 14;24:932. doi: 10.1186/s12913-024-11306-3 (PMC11325824; doi:10.1186/s12913-024-11306-3)
Supplement: Supplementary file 3 — Supplementary Material 3: Additional file 3 ConQual table [file 12913_2024_11306_MOESM3_ESM.docx]

**Additional file 3 – ConQual table**

**Summary of findings**

| **Systematic review title**: Experiences of informal caregivers supporting individuals with upper gastrointestinal cancers: a systematic review  Population: Caregivers providing unpaid care-work to patients of upper gastrointestinal cancers (UGICs)  Phenomena of interest: The experiences of informal caregivers and their role within the wider patient supportive setting  Context: Adult friends and family members providing care in the home setting and beyond for needs arising from UGICs | | | | | |
| --- | --- | --- | --- | --- | --- |
| Synthesised Finding | Type of research | Dependability | Credibility | ConQual Score | Comments |
| **UGIC caregiver burden** | Qualitative | Downgrades 1 level | Downgrades 1 level | Low | Dependability: More than half of studies (8 out of 17) scored 2-3 out of 5 for questions relating to appropriateness of the conduct of the research, therefore the dependability score downgrades 1 level. Seven studies scored 4, and two scored 5 out of 5.    Credibility: Downgraded one level due to mix of mainly unequivocal (U) and credible (C) findings. |
| **Mediators of caregiver burden** | Qualitative | Downgrades 1 level | Downgrades 1 level | Low | Dependability: The majority of studies (10 out of 18) scored 2-3 out of 5 for questions relating to appropriateness of the conduct of the research, therefore the dependability score downgrades 1 level. Six studies scored 4, and two scored 5 out of 5.    Credibility: Downgraded one level due to mix of mainly unequivocal (U) and credible (C) findings. |
| **Consequences of caregiver burden** | Qualitative | Downgrades 1 level | Downgrades 1 level | Low | Dependability: half of studies (8 out of 16) scored 2-3 out of 5 for questions relating to appropriateness of the conduct of research, therefore the dependability score downgrades 1 level. Six studies scored 4, and two scored 5 out of 5.    Credibility: Downgraded one level due to mix of mainly unequivocal (U) and credible (C) findings. |
