# Supplementary material for: Experiences of informal caregivers supporting individuals with upper gastrointestinal cancers: a systematic review
Source: BMC Health Serv Res. 2024 Aug 14;24:932. doi: 10.1186/s12913-024-11306-3 (PMC11325824; doi:10.1186/s12913-024-11306-3)
Supplement: Supplementary file 4 — Supplementary Material 4: Additional file 4 Characteristics of studies [file 12913_2024_11306_MOESM4_ESM.docx]

**Additional file 4 – characteristics of included studies**

| **Study** | **Method** | **Phenomena of interest** | **Participants** | **Author's conclusion** |  |
| --- | --- | --- | --- | --- | --- |
| Andreassen et al., 2005 Sweden | Semi-structured interviews were performed with family members either at the participant’s home, at the researcher’s office or at a hospital. | Family members’ experiences, information needs and information seeking in relation to living with a patient suffering from oesophageal cancer. | caregivers of oesophageal cancer patients were recruited from an ongoing study (n=9).  Age range 18+ | Family members were not aware of the severe diagnosis and the illness caused intrusions on the family. The time following diagnosis family members' information seeking was low. They used interpersonal as well as mass media sources to obtain knowledge about and handle the uncertainty related to the illness. Some family members did not actively seek information. |  |
| Gerdhardt et al., 2020 Denmark | Semi-structured, individual interviews were conducted with caregivers of patients in participants' homes. | The experiences of caregivers of patients attending the current routine follow-up aftertreatment with curative intent for PDB cancers (cancers in the pancreas, duodenum and bile duct). | caregivers of PBD cancer patients recruited from specialized gastro-surgical center at a tertiary hospital (n=10).  Age range = 36-82 | Caregivers described a feeling of transcending from bystanders to enlisted caregivers during the treatment pathway and follow-up. They experienced a tacit expectation from HCPs to perform a range of practical tasks during treatment and follow-up without feeling adequately in-structed or supported. Their needs were not systematically incorporated during the patient's treatment and follow-up. They dealt with their concerns on their own while stoically supporting and outwardly remaining positive towards the patient. This inferred an experience of isolation and distress in caregivers and may have affected mutual coping and emotional aspects in the relationship with the patient. |  |
| Gooden & White, 2013 Australia | Semi-structured interviews were conducted, telephone or face-to-face, exploring participants' caring perspectives and experience. | How pancreatic cancer is experienced from the different perspectives of those with a diagnosis and the caregiver/family to identify the areas of unmet psychosocial supportive care needs. | caregivers of pancreatic cancer patients from Australia Cancer Council Helpline or by direct clinician’s referral n = 23).  Age range 20-79 | The findings revealed that the major quality of life theme was difficulty in managing gut symptoms and complex dietary issues. Issues were related to lack of information about malabsorption and managing symptoms of pancreatic exocrine insufficiency. This was compounded by a lack of routine dietary consultation: perceived reluctance of clinicians to prescribe enzyme supplements and poor understanding of dose to diet guidelines. |  |
| Hansen et al., 2017 USA | In person semi structured interviews were conducted with caregivers once a month for six months. | Family caregivers’ perspectives about caring for patients with terminal hepatocellular carcinoma as the patients approached the end of life. | Caregivers of terminal HCC [LGW6] patients from the Oregon Health and Science University in Portland and Veterans Affairs Portland Health Care System (n=13).  Age range = 22-68 | Five core categories and nine subcategories were identified. From the time of the terminal diagnosis to the end of life, family caregivers felt unprepared, uncertain, and in need of information. They struggled with whether symptoms were HCC- or cirrhosis-related. |  |
| Hodgson, 2006 UK | caregivers completed questionnaires which included both open and closed questions to explore their caregiver experiences. | How nurses could improve the care offered to people following surgery for oesohageal cancer. | caregivers of oesophageal cancer patients from a group meeting with Oesophageal Patients Association (OPA) (n=9.  Age range = 18+ | Good quality of life is achievable for patients with and recovering from OC, but physical limitations and symptoms following surgery are not the only focus. Patients and caregivers put the focus on adaptation and accepting lifestyle changes, while remaining positive is essential and this is aided by the support from the OPA. |  |
| Larsen et al., 2020 Denmark | Two focus group interviews took place to explore caregivers experiences. | Relatives’ experiences with illness, treatment of the patient and decision-making in the context of Oesophageal Cancer. | caregivers of oesophageal cancer patients through patients receiving palliative oncological treatment (n=11).  Age range = 20-79 | The relatives were fellow sufferers, experiencing uncertainties and fear for the future with the patients, but they were simultaneously a challenged anchor during a difficult time, actively involved in handling the diagnosis and the everyday life. The relatives were positioned on the sideline both by the professionals and by themselves; they took a passive and subordinate part in decisions. |  |
| Larsen et al., 2021 Denmark | Participant observations and semi-structured interviews were used. | To explore relatives’ experiences before the start of treatment and their subsequent roles and needs for participation in treatment decisions. | caregivers of oesophageal cancer patients (n=19).  Age range = 20-79 | Relatives are central to cancer care and treatment. Adequate and timely information is imperative for relatives as well for patients in order to facilitate shared decision-making. We advocate for a new approach to relatives in order to prepare the relatives for their roles and support their individual needs but also to acknowledge relatives’ knowledge about everyday life from the relatives’ perspective. |  |
| McCorry et al., 2009 UK | Focus groups of Oesophageal Patients’ Association support group members took place at meetings, caregivers were separated from survivors. | Explore the cognitive and emotional experiences of esophageal cancer survivors, and those of their caregivers. | caregivers of oesophageal cancer patients from Oesophageal Patients’ Association (OPA) patients’ support group in Northern Ireland (n=10).  Age range 18+ | Esophageal cancer patients and their caregivers require holistic support in their efforts to adjust to the social, emotional and physical consequences of esophagectomy. Peers could be an effective channel for the support of patients and caregivers. |  |
| Morowatisharifabad et al., 2019 Iran | Semi-structured face to face interviews were conducted with caregivers. | Perceived threats by immediate relatives of patients with oesophageal cancer. | caregivers of oesophageal cancer patients recruited among attendees of governmental public health centres (n=23).  Age range = 20-75 | Immediate relatives of patients with esophagus cancer perceived high severity and susceptibility toward this disease and considered themselves vulnerable to this disease. Hence, they suffered from a lot of psychological stress. Development of appropriate interventions regarding the effects of disease-related individual and environmental factors and empowerment of individuals regarding preventive disease behaviors can be an important step for improving the health of this group. |  |
| Morowatisharifabad et al., 2020 Iran | Qualitative semi-structured interviews were used to explore caregivers experience. | To elaborate on the concerns and fear of esophageal cancer in relatives with cancer illness. | caregivers of oesophageal cancer patients from attendees at governmental centres (n=23).  Age range = 20-75 | Family of patients with esophageal cancer were afraid of disease-induced consequences and lack of knowledge about the disease. Through educating people about the symptoms of the disease, we can reduce their fears and empower them for self-caring and preventing cancer. |  |
| Nolan et al., 2006 USA | Identification of categories and themes in Web postings using the constant comparison method of content analysis. Main Research Variables: Spirituality, relationship of the person posting a message (poster) to the person with cancer. | The spiritual issues addressed in the patient and family chat room of a pancreatic cancer Web site. | Caregivers of pancreatic cancer patients posted writings to an online chat room of Johns Hopkins Hospital’s pancreatic cancer Web site which was sampled (n=600 internet postings) | Many individuals, particularly the family caregivers of patients with pancreatic cancer, are able to express their spirituality and find spiritual support in the chat room of a Web site devoted to the disease. Nurses and other healthcare professionals who develop and evaluate cancer information Web sites can take a more holistic view of users by supporting their spirituality along with providing in-formation on disease diagnosis, management, and prognosis. Further study is needed to identify facets of cancer information Web sites that can provide spiritual support to individuals and facilitate the development of supportive cancer Web site communities. |  |
| Padron et al., 2018 USA | Participants took part in group discussion exploring caregiver’s experience. | To examine the unmet needs of peripancreatic cancer patients and their caregivers through the implementation of Photovoice. | caregivers of peripancreatic cancer patient from the UF Hematology-Oncology Clinic (n=9).  Age range 18+ | Female patients reported unmet psychosocial needs despite their male partner/caregivers use and belief in the effectiveness of problem-focused coping to manage dyadic distress. |  |
| Petrin et al., 2009 USA | Trained clinical interviewers and a genetic counsellor conducted telephone interviews with participants on their caring perceptions. | Examine family communications about a family member’s diagnosis of pancreatic cancer, treatment, and his or her survival or death. | caregivers [LGW3] of pancreatic cancer patients from the Cancer Genetics Network (n=22).  Age range 18+ | Pancreatic cancer families may be unable to cope by taking one day at a time and must find other ways of dealing with stress. Also, the compressed timeline between diagnosis and death may heighten certain coping behaviours, such as the revaluation of one's priorities. |  |
| Shaw et al., 2013 Australia | Semi-structured telephone interviews were conducted at 3 weeks and 3 months post-surgery. | To explore the experiences of family caregivers of people diagnosed with upper GI cancer after surgical intervention to (1) identify their unmet supportive care needs and (2) investigate how family caregivers perceive their role during this time. | Caregivers of post-surgical upper GI cancer patients recruited from surgery lists in two metropolitan hospitals (n=15).  Age range 18+ | This study provides new insight into the supportive care needs of family caregivers of upper GI cancer patients and the impact of unmet need on the emotional well-being of family caregivers. These results will inform future supportive care service development and intervention research aimed at reducing unmet supportive care needs and psychological distress of family caregivers of patients with poor prognosis upper GI cancer. |  |
| Sherman et al., 2014 USA | Semi structured telephone and in-person interviews were used, informed by the Stress Process Model, were carried out exploring participants caring experiences. | The experience of family caregivers of patients with advanced pancreatic cancer. | caregivers of advanced pancreatic cancer patients recruited from oncology practices of a university-affiliated medical centre (n=8).  Age range = 37-74 | Findings indicated caregivers' willingness to participate in research, highlighted the negative and positive aspects of the caregiver experience, and reinforced the significance of the future study and the need to develop interventions to support family caregivers in their roles. |  |
| Shih et al., 2013 Taiwan | Face to face semi-structured interviews were used to collect information regarding the family’s subjective experience. | To explore the experiences of families of patients with newly diagnosed advanced terminal stage hepatocellular cancer. | Caregivers of terminal HCC cancer patients were recruited in this study (n=9).  Age range 18+ | development and intervention research aimed at providing assistance in reducing unmet supportive care needs and psychological distress of these family members. |  |
| Winterling et al., 2004 Sweden | Semi structured interviews took place with caregiver spouses in person either in their home or in hospital. | Perceptions of changes in life among patients who have recently been diagnosed with an advanced gastrointestinal cancer and among their spouses. | caregiver spouses of advanced GI cancer patients recruited from attendees at an oncology department (n=14).  Age range = 43-83 | More patients than spouses seemed to accept their situation because fewer patients complained and instead prepared for death, whereas more spouses felt despair, used hope and avoidance, and were preoccupied with practical matters. These findings suggest that spouses are a vulnerable group and healthcare staff should be just as aware of their situation as that of the patients |  |
| Wong et al., 2019 USA | Using photovoice methods, participants took photographs and then provided group discussion narratives depicting their caring experiences. | Factors of pancreatic cancer associated with psychological distress from patient and caregiver perspectives. | caregivers of pancreatic cancer patients from an academic teaching hospital (n=7).  Age range 50-69 | Commonalities between patient and caregiver sources of distress emerged despite their distinct roles. Findings revealed four major areas of distress: diagnosis of an unexpected, advanced cancer, changes in roles and identity, management of weight loss and gastrointestinal problems, and fear of the future. Participants also discussed unique perspectives such as the stigma of pancreatic cancer and caregiver guilt. |  |
| Yi & Kahn, 2004 South Korea | This was a secondary analysis study using grounded theory techniques. The study used the data collected from in-depth interviews from two primary studies. | To explore the experiences of gastric cancer couples in Korea and to generate a substantive theory integrating the experiences of gastric cancer survivors and their spouses. The specific aims of this study were to explore major problems gastric cancer couples faced and how they resolved these problems, focusing on inter-relational dynamics within the couples and on similarities and differences between the couples. | caregiver spouses of gastric cancer patients contributed to a primary data set which was drawn from (n=11).  Age range = 18+ | The results of this study will help design family care for the people with gastric cancer by providing in-depth understanding and insight on the lives of gastric cancer couples. |  |
