# Supplementary material for: Experiences of informal caregivers supporting individuals with upper gastrointestinal cancers: a systematic review
Source: BMC Health Serv Res. 2024 Aug 14;24:932. doi: 10.1186/s12913-024-11306-3 (PMC11325824; doi:10.1186/s12913-024-11306-3)
Supplement: Supplementary file 5 — Supplementary Material 5: Additional file 5 Methodological assessment [file 12913_2024_11306_MOESM5_ESM.docx]

**Additional file 5 - Results of methodological assessment of included articles**

|  |  | **Question** | | | | | | | | | |  |
| --- | --- | --- | --- | --- | --- | --- | --- | --- | --- | --- | --- | --- |
|  | **Citation** | **1** | **2*** | **3*** | **4*** | **5** | **6*** | **7*** | **8** | **9** | **10** | % |
| 1 | Andreassen *et al.* (2005) | Y | Y | Y | Y | Y | N | N | Y | Y | Y | 80% |
| 2 | Gerhardt *et al,* (2002) | Y | Y | Y | Y | Y | N | Y | Y | Y | Y | 90% |
| 3 | Gooden *et al*, (2013) | Y | Y | Y | Y | Y | N | N | Y | Y | Y | 80% |
| 4 | Hansen *et al,* (2017) | Y | U | Y | Y | Y | N | N | Y | Y | Y | 70% |
| 5 | Hodgson. T, (2006) | Y | Y | Y | Y | Y | N | Y | Y | N | Y | 80% |
| 6 | Larsen et al, (2020) | Y | Y | Y | Y | Y | Y | Y | Y | N/A† | Y | 90% |
| 7 | Larsen *et al,* (2021) | Y | Y | Y | Y | Y | Y | Y | Y | Y | Y | 100% |
| 8 | McCorry *et al*, (2009) | U | Y | Y | Y | Y | N | U | Y | Y | Y | 70% |
| 9 | Morowatisharifaba *et al*, (2020) | Y | Y | Y | Y | Y | Y | N | Y | Y | Y | 90% |
| 10 | Morowatisharifaba *et al*, (2021) | Y | Y | Y | Y | Y | Y | N | Y | Y | Y | 90% |
| 11 | Nolan *et al,* (2006) | Y | Y | Y | Y | Y | N | N | Y | Y | Y | 80% |
| 12 | Padron. A, (2018) | Y | Y | Y | Y | Y | N | N | Y | N | Y | 70% |
| 13 | Petrin *et al,* (2009) | U | Y | Y | Y | Y | N | N | Y | U | Y | 60% |
| 14 | Shaw *et al,* (2013) | Y | Y | Y | Y | Y | N | U | Y | Y | Y | 80% |
| 15 | Sherman *et al,* (2014) | Y | Y | Y | Y | Y | N | Y | Y | N | Y | 80% |
| 16 | Shih *et al,* (2013) | Y | Y | Y | Y | Y | N | Y | Y | N | Y | 80% |
| 17 | Winterling *et al,* (2004) | Y | Y | Y | Y | Y | N | N | Y | U | Y | 70% |
| 18 | Wong *et al,* (2019) | Y | Y | Y | Y | Y | N | N | Y | Y | Y | 80% |
| 19 | Yi *et al,* (2004) | Y | Y | U | Y | Y | Y | Y | Y | N/A† | Y | 80% |
|  | % | 89% | 95% | 95% | 100% | 100% | 26% | 37% | 100% | 58% | 100% |  |

Y, Yes; no, No; U, Unclear; N/A, Not Applicable. * Dependability questions.

† N/A rating given as authors stated that study^15^ had been conducted on secondary data, and study^22^ stated that it had been conducted in accordance with the Declaration of Helsinki and Danish Data Protection Agency.

| 1. Is there congruity between the stated philosophical perspective and the research methodology? |
| --- |
| 1. Is there congruity between the research methodology and the research question or objectives? |
| 1. Is there congruity between the research methodology and the methods used to collect data? |
| 1. Is there congruity between the research methodology and the representation and analysis of data? |
| 1. Is there congruity between the research methodology and the interpretation of results? |
| 1. Is there a statement locating the researcher culturally or theoretically? |
| 1. Is the influence of the researcher on the research, and vice- versa, addressed? |
| 1. Are participants, and their voices, adequately represented? |
| 1. Is the research ethical according to current criteria or, for recent studies, and is there evidence of ethical approval by an appropriate body? |
| 1. Do the conclusions drawn in the research report flow from the analysis, or interpretation, of the data? |
