# Supplementary material for: Experiences of informal caregivers supporting individuals with upper gastrointestinal cancers: a systematic review
Source: BMC Health Serv Res. 2024 Aug 14;24:932. doi: 10.1186/s12913-024-11306-3 (PMC11325824; doi:10.1186/s12913-024-11306-3)
Supplement: Supplementary file 6 — Supplementary Material 6: Additional file 6 Meta-synthesis [file 12913_2024_11306_MOESM6_ESM.docx]

**Additional file 6 – Meta-synthesis**

Meta-synthesis 1 – UGIC caregiver burden

| **Findings (n=80)** | **Categories (n=5)** | **Synthesised Finding** |
| --- | --- | --- |
| (A5) Burden of responsibility for patient’s recovery [U]  (B11) Feeling of being able to contribute in some way was helpful. [U]  (B9) Family members felt responsible for helping the patient by taking care of scheduling and attending doctor’s appointments and treatments, activities seemed to be to take one’s mind off of the emotional handling of the illness. [U]  (E15) the focus on the patient was all encompassing. These participants adopted sole responsibility for patient care. [U]  (E20) Perceived as meeting patient physical needs, and the multifaceted responsibility of providing support to the patient. [role] [C]  (F10) This devotion to cooking had made female spouses physically drained because most survivors expected to eat newly cooked food at every meal [U]  (F14) Spouses focused more on “reinforcing physical strength” to get the survivors well [C]  (F7) The spouses devoted their entire energy into cooking, providing the best freshest food to the survivors at every meal [U]  (I8) The family caregiver may be the primary caregiver, who assumed total responsibility on a 24 hours basis. [C]  (I24) Caregivers, however, were ‘‘wanting to be helpful’’ and ‘‘take off the pressure,’’ which created a sense of resentment on the part of the patient. [U]  (K17) They saw the treatment schedule as a joint event, supporting the patient to remember medication at the appointed hours. [U]  (K23) [Relatives are] undertaking a huge responsibility, and constantly alert to whether the patients lost or gained weight. [U]  (L6) caregivers felt like they were one person trying to assume five different roles of a nurse, secretary, and pharmacist, all while maintaining who they were and picking up the patient’s previous tasks. [C]  (M15) spouses had to organize and carry out almost all the practical things that needed to be done... The spouses said that they wanted to help the patient because it was their duty as a partner. [U]  (O10) Participants described disease-related problems in their patients, including physical problems and apparent changes due to illness [U]  (P12) Properly supervised EST did show stabilisation of or increase in weight, resolution of pain, diarrhoea and abdominal discomfort [U]  (R16) As the disease advanced and symptoms became more pronounced, family caregivers interpreted visible changes (e.g., sunken eyes, difficulty walking) as signs of worsening condition and markers of disease stage. [C]  (S5) Caregivers consequently felt they were silently enlisted as carers of the patients both during hospitalization and after hospital discharge. Their “job” included care of surgical drains, surgical wounds, nutritional and gastrointestinal function and postoperative pain management. [U] | **Breadth of caregiver role**  Participants expressed an obligation to undertake new responsibilities as care-provider to make a concrete contribution in helping the patient adjust to the UGIC diagnosis, regardless of their preparedness. *“Feeling of being able to contribute in some way was helpful.”* (B11) | **Synthesised finding 1 – UGIC caregiver burden:**  As caregivers began supporting those with UGICs, numerous adjustment and challenges were encountered. Individuals learnt to adjust to their caregiver role within their existing lives and to the different elements of patient care required specifically in relation to UGICs which included challenges around patient mealtimes. These difficulties impacted caregivers’ psychological health. |
| (A7) Carers' representations of food and eating were heavily emotionally laden and they still represented recovery in terms of the ability to eat larger quantities. [C]  (E12) Information related to patient nutrition was of particular concern to family caregivers at both time points and contributed to family caregiver distress. [U]  (H12) Eating was seen as a unifying family ritual. Thus, when someone in the family was unable to eat due to dysphagia, loss of togetherness and social life was evident. [U]  (K21) Especially the burdensome task of eating was perceived as a mutual struggle [U]  (K22) Troubles with eating became a joint concern, and relatives were deeply engaged with adjusting to new eating habits [U]  (K24) The worry about patients’ weight was, largely, associated with patients’ progress and recovery, making meals into events burdened with anxiety and despair. [U]  (P13) For the carer/family group, concerns around managing diet and symptoms of PEI were linked to increased carer burden and significant distress. Participants expressed feelings of anger, frustration and powerlessness, as they struggled to find foods for their loved ones to eat that minimised symptoms. [U]  (P14) It was also flagged as causing the most tension and difficulties in relationships between carer and patient in the dyad subgroup [U]  (P2) Participants talked about experiencing symptoms of fat malabsorption and steatorrhoea [U]  (P5) Problems with diet were compounded by a lack of access to dieticians or routine assessment in the clinical setting and/or follow-up post-discharge [U]  (Q1) They identified how life revolved around food [C] | **Challenges around patients’ meals**   There is a high emotional significance around eating and meals*, “We find it difficult to figure out what he can eat.”* (K21), which is compromised due to the nature of UGICs. The distress is shared between both caregiver and receiver. |  |
| (B5) Pancreatic cancer diagnosis as having a significant impact on their lives. [C]  (B10) Need to manage aspects of his own life [C]  (B13) If visiting was not an option, because of a physical distance, for example, this was a source of significant stress. [U]  (C12) Relatives felt their daily lives collapsing and they lost control. [C]  (E17) these participants reported that there was little time to consider their own need for support as they were trying to meet the needs of others. [C]  (E18) Others reported having to balance their care for the patient with other pre-existing caring and work responsibilities. [U]  (H11) It was found that the cancer became intrusive in the family’s life and routines [U]  (I33) The caregiver’s own social life became limited [U]  (J3) felt their lives were seriously disrupted by their loved one’s illnesses, which resulted in increased responsibilities, rearranged priorities, and an inability to plan. [U]  (K18) [scheduling around medication and appointments] could be experienced as a strain on and disruption of everyday life [C]  (K19) Because of the treatment schedule, relatives experienced a restricted daily life, adjusting to new routines [U]  (M8) Informants felt that this was unfair since they already had another serious illness that influenced their life, that there were other people who were older and healthier, or felt disappointed at not being able to do things they planned for [C]  (O17) Enduring great difficulty during treatment [due to distances]. [C]  (O18) Long-term treatment of the disease [U]  (O19) Persistent medical check-up [U]  (O20) Conducting surgery and removing tumor [U]  (O21) Cancer as a life disturbance [U]  (R9) A terminal HCC diagnosis brought a change in lifestyle, particularly as patients underwent treatments or required full-time care. [U] | **Life disruption**  Individuals are often leading full lives before they are faced with the additional role of caregiver. This new role often disrupts prior commitments and priorities for caregivers, *“we can’t live a normal life’* (H11). |  |
| (C6) did not get time to reflect about the new life situation [U]  (C7) the speed in diagnosing made them feel behind in the schedule. [U]  (D6) Fear of impatience to treatment [U]  (E7) understanding of the surgery but were unprepared for the level of patient care required. [U]  (C28) they were unfamiliar with the treatment and that it could be difficult to know which questions to ask. [U]  (D9) lack of information about the disease. [U]  (E10) lack of specific information was exacerbated when, prior to discharge, family caregivers were not present during discussions with the treating team [U]  (E21) Lack of information regarding the availability of financial and com-munity support resulted in additional stress for family caregivers; little awareness regarding the role of the hospital social work team in facilitating support and few had met with a social worker. [U]  (H2) Due to the vague symptoms of oesophageal cancer as well as the patient’s lack of awareness of the disease, family members in this study reported that there was a delay in consulting the physician with the symptom. [U]  (H28) not asking questions was due to their lack of medical knowledge about oesophageal cancer. [U]  (H30) Concerning oesophageal cancer, they were ignorant and had never heard of the disease. [U]  (H38) there was an oscillation between family members’ desire for more information and the avoidance of new information. [U]  (I12) Family caregivers discussed openly either a willingness to care born out a sense of responsibility or duty or (less often) a sense of ‘‘being forced’’ to assume caregiving responsibilities, with a perception of great personal sacrifice. [C]  (I17) lack of clear information and support from health professionals was often an intense source of distress.  (L5) The sudden adjustment to learning new medical information ... [U]  (P15) Participants’ lack of knowledge about the effects of pancreatic disease on eating and digestion meant some of them continued struggling with symptoms, in ignorance [U]  (P16) For the bereaved group, it was this particular issue that stood out for them as having increased their distress contributing to their feelings of unresolved grief. [C]  (R17) Family caregivers frequently spoke about a lack of information from HCPs regarding treatments and their side effects. [U]  (B8) Feeling that things were happening quickly, and that time was of the essence [U]  (E8) family caregivers perceived that they lacked the knowledge to meet all the patient’s needs and were fearful of providing inadequate care. [C]  (R15) Without an understanding of HCC and its potential progression to liver failure, family caregivers had difficulty identifying what was happening and how to respond to unexpected complications. Hepatic encephalopathy was particularly challenging. [U]  (R19) Although most family caregivers were aware that the patient in their care would eventually die, some felt unprepared to provide end-of-life care themselves and continued to lack information about what to expect. [U]  (R20) As patients with a history of substance abuse neared end of life, pain management continued to be challenging and difficult for family caregivers to understand [U]  (R21) One non-spousal caregiver was surprised to learn that she would be responsible for providing such care. [U]  (R5) All family caregivers reported having little knowledge of HCC and little to no knowledge of providing care to people with terminal HCC [U]  (S6) They described doing so without adequate instruction and minimal interest from HCPs as to whether they performed the tasks correctly. [U] | **Unpreparedness**  Coming from a non-medical background many caregivers are thrust into a new role with unanticipated role demands that they feel ill-prepared for, “don’t think they gave us enough information about how to look after him” (E8) |  |
| (A6) Carer was also a conduit who provided explanations to family and friends [C]  (B17) the importance of communicating with family members, often with the ill individual him- or herself. [U]  (C1) relatives were generally present in the patients’ consultations with health professionals; Relatives could add important information in consultation [U]  (C2) relatives often put into words their view on how the patient managed everyday life [U]  (I18) revealing or concealing the diagnosis and the prognosis to patients. [U]  (I32) Family caregivers were the conduit of medical information to other family members [U]  (S14) Although caregivers themselves were anxious and fearful of disease recurrence, especially if patients developed worrying physical symptoms, they kept their anxiety and fears to themselves. [U] | **Information manager**  Caregivers were often responsible for communicating information between different parties such as the patient, *“hope would be taken away if she knew”* (I18), healthcare providers and other family members. Equally they managed which information should not be shared e.g. a poor prognosis hidden form the patient. |  |

Meta-synthesis 2 – Mediators of caregiver burden

| **Findings (n=130)** | **Categories (n=6)** | **Synthesised Finding** |
| --- | --- | --- |
| (C21) should not be part of decisions, and it was natural that health professionals did not invite them to participate. [U]  (C22) relatives took a subordinate part in consultations, as they considered the consultation to be owned by the patient. [U]  (C23) might have questions themselves, which they did not always get the permission to ask. [U]  (C24) Relatives and patients were not always in agreement about which questions to ask, which made it difficult for relatives; they were left with unanswered questions. [U]  (C25) amount of information they received from health professionals was large, but they also acknowledged the need for an overview of the treatment pathway [U]  (C28) sometimes they chose not to ask questions about the future as they relied on the authority of health professionals. [U]  (C3) relatives were not always invited to be an active part of the consultation by the health professionals [U]  (C4) observations showed that relatives often did not have an established role in consultations but were merely looked upon as an appendage to the patients leading to relatives were positioned on the sideline and not anchored in consultations. [U]  (H10) family members experienced that healthcare professionals focused on the care of the patients, which made the family members feel ‘invisible’. [U]  (H27) all of the family members did not want to discuss and ask specific questions with the physician when the patient listened. [U]  (I2) caregivers expressed feeling ‘‘lost in the health care system.’’ [U]  (I3) The health care system offered little help in navigating the system, [U]  (K32) Relatives did not think they should be active in the hospital context. [U]  (K33) They took a passive position, not asking questions, awaiting the directives of health professionals. [C]  (K34) Some relatives expressed a lack of courage to pose questions [U]  (K35) Relatives positioned themselves on the sideline, subjected to the authority of both the healthcare professional and the patient. [U]  (K36) They depended on patients and healthcare professionals to give them space and acknowledge their roles as relatives. [U]  (K37) Some relatives stated that they needed to call attention to themselves. [U]  (K38) Relatives did not feel rejected, but they reflected on the missing invitation to be part of the treatment course and decision-making. [C]  (K39) If relatives were to participate, they needed to authorise themselves, making themselves visible and claiming their roles as relatives. [U]  (K40) The missing empowerment by patients and health professionals caused relatives to have unanswered questions. [U]  (L8) lack of [direct] interactions with medical providers [C]  (P9) This distress was exacerbated by a perceived reluctance of clinicians to prescribe EST and the lack of understanding as to the need for management of PEI. [U]  (S13) Some patients wished to attend follow-up visits on their own without the caregiver present. This made it more difficult for caregivers to keep tabs on the patient's situation and provide adequate support. [C]  (S3) HCPs seldomly approached caregivers proactively to inquire specifically about their needs. [C]  (S4) HCPs were nevertheless always friendly and accommodating when caregivers themselves took the initiative to discuss or ask HCPs questions [C]  (S18) Caregivers reflected that, in hindsight, they potentially could have benefitted from talking to someone who was not personally involved in the disease trajectory, a professional of some sort. [C] | **Degree of inclusion in medical settings**  Caregivers reported that HCPs focused their attention solely on the patient often to the point of excluding the caregiver, *“Even when you go in and see Dr. X or any of the other providers, it’s about them [the patients]. Nobody says how are you doing?””* (L8).  Patients also often left caregivers out, making caregivers feel uninformed. | **Synthesised finding 2 - Mediators of caregiver burden:**  While supporting patients with upper GI cancer caregivers are exposed to mediators which influenced their caregiver experience, including coping, resources, and their caregiver context. For instance, how involved a caregiver was in the medical setting influenced the caregiver burden experienced. |
| (A10) Recognized the value of peer support, especially for normalization of experiences reducing feelings of isolation, and a source of hope [U]  (B16) The degree to which individuals felt they received support and sources of that support varied. [C]  (B18) took comfort in being able to talk to friends, particularly those who had a similar experience of dealing with an ill family member [C]  (B19) others sought solace in the church, both in attending church services as well as engaging in religious observance in private. [C]  (B21) others were hampered by a lack of family communication or more formal types of social support. [U]  (E22) A pre-existing relationship with a GP was a major determinant of ongoing practical support. [C]  (F37) Support from their spouses was necessary in [helping change survivors’ value systems to accept modest life according to Taosim, Buddhism or Christianity] [C]  (H22) the family members also felt connected to the nurses who could answer questions of importance and give practical and emotional support. [U]  (I6) Several participants spoke about how others enhanced or interfered with interactions with health care professionals given their own expectations,  (I9) Alternatively, a caregiver may assume secondary responsibilities for caregiving, acting as support when the primary caregiver needs relief, or there was a sharing of responsibilities with other family members. [U]  (I10) Often, physical proximity to the patient resulted in expectations by patients, caregivers themselves, or extended family that the member in closest physical proximity would assume a direct caregiving role. [U]  (I34) Caregivers related that ‘‘friends don’t know what to say’’ or even worse ‘‘just don’t ask’’ and that really hurts [U]  (N1) Promises of prayer and requests for prayer were some of the most common spiritual issues observed in the postings [U]  (N14) postings reported the death of a loved one with pancreatic cancer. [U]  (N2) A number of posters acknowledged that their fellow chat room contributors may have diverse spiritual and religious practices [U]  (N3) Several posters shared with others the blessings for which they were thankful [C] | **Social resource**  As the diagnosis of an UGIC comes with emotional upheaval and major practical adjustments, the absence or presence of support was crucial to the caregiving experience. |  |
| (C13) fears about their economic situation and had a need to establish an overview of the situation [C]  (D5) fear of financial problems and burdens. [U]  E16) given up activities to support the patient, including work outside the home [U]  (J5) difficulty in planning ahead since cancer is a severe disease to affect their lives [U]  (K6) This caused reflections about economy and practical issues [U]  (K28) Claiming control seemed to be part of coping with a changed life situation. Knowing that they could manage areas such as expenses made them more at ease with the threat of cancer. [C]  (R10) Some spousal caregivers stopped working to provide full-time care and had a difficult time adjusting to the change. [C] | **Financial resources**  As UGICs tend to be financially and labour intensive, logistical concerns had to be solved and this caused stress. |  |
| (I7) Within the context of the patient/ caregiver relationship, there were varying ages and degrees of emotional closeness dependent on the history of the relationship. [U]  (B22) Family dynamics, conveying either implicitly or explicitly that whatever underlying foundation had existed prior to the diagnosis remained intact. [U]  (B24) some participants coming from families that were distant harboured resentment over being burdened with caring for the ill family member. [U]  (E19) family caregivers did not perceive themselves as Carers. Providing care was viewed as a natural extension of the family relationship [U]  (F12) When preparing folk medicine the spouses became a curer rather than a carer for the survivors, thinking that their partners’ live relied totally on them [U]  (F22) It made spouses disappointed and frustrated, and in the end, some even reproached the survivors for getting the cancer [U]  (F35) The relationship between the spouses became unstable, even for those who had no marital problems before. They became overly sensitive and annoyed at each other. [C]  (G1) male caregivers derived a sense of control over their lives and environments through concrete problem-solving. [C]  (H23) the patients themselves were considered experts. [U]  (I21) the family caregiver and other family members experienced reciprocal suffering [U]  (I56) a shift of focus from society, the community, and family to a laser-like focus on the patient and relationship. [C]  (K27) The former relationship between relatives and patients could change. The changed life situation caused relatives to reflect on the distribution of roles within the family. [U]  (K29) A changed balance within the family, and sometimes wives thought they took a motherly role towards their patient husband. [U]  (O22) Participants acknowledged that patients regarded themselves as being a burden on their families. [U]  (R2) Most non-spousal caregivers described having an emotionally distant, non-supportive relationship with the patient. [C]  (R3) Underscoring some relationships was the patients’ history of substance abuse and family conflict. [C] | **Patient-** **caregiver relationship**  The experience of caring was often shaped by the quality of relationship shared between caregiver and patient*. “Family dynamics, conveying either implicitly or explicitly that whatever underlying foundation had existed prior to the diagnosis remained intact.”* (B22) |  |
| (A1) Responsibility for protecting the patient and their family from distress [withhold information] [U]  (A2) Needing to be strong for those around them [U]  (B20) Those who coped in this way found it to be helpful in sorting out their feelings and in dealing with them. [Therapy included counselling and in one instance, a course of antidepressant therapy] [C]  (C15) create a refuge from the disease and balance everyday life. [U]  (C8) feel obliged to inspire confidence in the patient regarding future and put up a facade. [U]  (F33) The spouses thought that they just had to absorb their complaints like a sponge [from their survivor spouse] [U]  (H31) the family members believed that the image of cancer given in Swedish mass media is that the survival rates are increasing. [U]  (H36) When the family members confronted the physicians with information about the prognosis of oesophageal cancer, they found that their reaction was positive. [U]  (I14) As the patient’s needs increased and the final days approached, with more family members in attendance, family caregivers felt an invasion of their personal space and a lack of privacy. [U])  (I20) the caregiver served as protector of not only the patient but also other family members who could not accept and face the death of someone they love. [U]  (I31) caregivers mirror the courage of the patient when they are really [falling apart] [C]  (I41) Several participants cited laughter and humour as ways of coping. [U]  (I42) Coped by for example “getting good news”, “denial”, “choosing not to think about it and “choosing what to hear” [C]  (I43) Participants also compared their experience with other patients and caregivers with advanced pancreatic cancer, particularly those ‘‘who were sicker or who died.’’ This engendered a ‘‘sense of gratitude’’ that ‘‘things could be worse.’’ [C]  (I44) What was important to all participants was “living in the present” and “looking for the good in every situation.” [U]  (I45) Speaking to ‘‘positive people’’ gave them strength and offered a supportive perspective. [U]  (K30) The position as buffer entailed the relative’s serving as a target for patients’ frustrations, trying to be supportive and optimistic. [U]  (M3) A few spouses stated that they thought that their partners would be cured. [U]  (M23) Some said that they tried to keep their spirit up. [C]  (M21) Few expressed a kind of confidence that everything would come out all right. [C]  (M22) One should not give up, but look ahead, be strong, and have a positive outlook. [U]  (M26) A few expressed the hope that the patient would be cured. [U]  (N10) expressed more specific hopes [C]  (N4) Some postings described feelings of conflict between spiritual beliefs and the present experience of suffering but seemed to present the conflict to other posters in an open-minded or hopeful way. [C]  (N8) Several posters cast the suffering of their loved ones in a more positive way [C]  (N9) For some, hope seemed all encompassing [U]  (N11) their loved ones were dying but hoped that their pain could be controlled during their last months of life [C]  (O14) Need high spirits to endure disease [U]  (S15) Caregivers made it clear that their responsibility was to “stick with the patient”, be positive, put their own feelings aside and their lives on hold to care for and keep the patient's spirits up, what we call “keeping a stiff upper lip”. [U] | **Emotion-oriented coping**  Due to the degree of distress caused by the life change, individuals coped by regulating their emotions and *“found it to be helpful in sorting out their feelings and in dealing with them”* (B20). |  |
| (B6) Information seeking was one of the most common coping-related themes. [U]  (B7) Wanted to know how long their loved one could be expected to survive, how severe the symptoms would be, and what treatments were available [U]  (C20) Knowing there was the possibility of treatment was a comfort and a relief. [U]  (C26) Knowledge of what to expect and how to manage the effects of treatment made relatives feel more at ease with the whole situation. [C]  (C27) It was important that health professionals were honest in their communications. [U]  (C5) relatives had an awareness of the severity of the disease [U]  (H21) the family members entrusted themselves to the experts, i.e. the physicians, who were considered the major source of information [U]  (H29) The family members contacted persons in the family’s circle who had specific knowledge of the illness and in whom they felt confidence. [C]  (H32) The family members looked in encyclopaedias, medical books, material produced by the hospital, and brochures, to gain medical information about the illness and to get an overview of problems related to the illness. [U]  (H33) Family members did not only seek information in order to gain increased medical knowledge, but also because it gave them the feeling of doing something constructive. [U]  (H34) used the Internet mainly to obtain an overview about the illness and illness-related problems as well as about the prognosis of oesophageal cancer. [U]  (H39) On the other hand, knowledge about details relating to the illness could alleviate some of the scariness and unpleasantness. [U]  (H40) Seeking information was sometimes considered as an effort for the family members, which demanded a considerable amount of time, courage and energy. [U]  (H5) The family members experienced that the physicians tried to counterbalance hope and honesty by using words like ‘cell changes’ (dysplasia) when giving the diagnosis of oesophageal cancer. They interpreted the physicians’ wording with a life-threatening disease. [U]  (H6) Not only the physicians’ vocabulary but also their body language was interpreted carefully [U]  (I19) caregivers expressed a need to understand the whole process. [U]  (I55) The greatest transition was when it was clear that the patient’s symptoms resulted from the disease rather than the treatment. [C]  (J7) participants wanted to search all possible regimens, such as Chinese herbs, healthy supplements, certain foods, religion, etc. [U]  (M10) Another wondered if the illness was some sort of punishment. [C]  (M12) Brooding about “why” also included wondering about the origin of the cancer. [U]  (N5) Two posters questioned why God wanted their loved ones to suffer. (C)  (O1) The role of individual performance in incidence of disease [U]  (O3) Prevalence of the disease due to living area [U]  (O4) people stated that our province has more rural areas because of the good climate and soil fertility conditions and people are often busy with many activities. [U]  (O5) Tensions … of life. [U]  (O6) ….pressures of life. [U]  (O8) Even participants complained about that food products that are available in the market [U]  (O9) Low monitoring of food available in the market [U]  (O11) The emergence of other diseases during chemotherapy [U]  (O12) Metastasis of the disease to other parts of the body [U]  (P10) Participants described how persistent and proactive they needed to be in accessing information and advice and how they had to ‘keep asking’ or ‘go elsewhere’ to access effective management for their symptoms. [U]  (P4) Did utilise the internet to source information about diet and gastrointestinal symptoms and it was online that they first found out about pancreatic exocrine insufficiency (PEI) [U]  (R14) As HCC progressed to liver failure, family caregivers were uncertain whether symptoms were related to liver failure or substance abuse. [U]  (R6) Many described receiving little information from HCPs and seeking out information from alternative sources (e.g., the Internet, books) to learn about HCC, symptoms, and treatments. [U]  (S12) caregivers sought to gather information about the disease from other sources, mainly from the internet. They kept their findings to themselves without passing them on to the patient. [U] | **Information seeking**  Caregivers found comfort in acquiring information about the disease aetiology, presentation, and management, and turned to online, offline and interpersonal sources. *“…relatives had an awareness of the severity of the disease”* (C5) |  |

Meta-synthesis 3 – Consequences of caregiver burden

| **Findings (n=99)** | **Categories (n=5)** | **Synthesised Finding** |
| --- | --- | --- |
| (B27) One individual, however, expressed the opposite sentiment [felt did not matter, taking care of body] [U]  (B31) Family members of patients who succumbed to pancreatic cancer described a challenging experience. [U]  (E1) This series of interviews highlighted the emotional burden of caring for UGIC patients post-surgery. Across interviews, family caregivers described their stress and anxiety. [U]  (E4) participants reported that they were physically and emotionally exhausted [U]  (E6) Family caregivers described being distressed by the cancer diagnosis and the impact of surgery on the patient. [C]  (F1) The gastric cancer couples considered diagnosis of gastric cancer as receiving “a death sentence.” [U]  (H4) Negative feelings were expressed in relation to the delay in diagnosis [U]  (I23) caregivers became extremely distressed by a sense of lack of control and often a sense of failure. [as disease progressed and death takes hold] [U]  (J8) A number of family members indicated their feelings of helplessness and yet they tried to search all kinds of possible regimens. [U]  (J9) when doctor announced patient’s diagnosis as newly diagnosis advanced terminal stage hepatocellular cancer, they felt unbearable and were filled with dismay. [U]  (K25) If the patient was struggling, that could destroy hope for progress [C]  (M1) Many patients and spouses were aware of the poor prognosis but some seemed to believe that there was a chance of a cure [U]  (K26) Some relatives could not bear to see the patient’s struggle [U]  (L2) Caregivers watched from the side-lines as they too felt helpless in their inability to control treatment or disease side effects [U]  (N7) Two others also asked God to take their loved ones [C]  (M6) Many spouses stated that they suffered when they saw the patients suffer from fatigue, pain, etc. [U]  (O15) Hate life due to pain and discomfort [U]  (N12) the concept of “false hope.” [C]  (O13) Psychological problems after being the disease [U]  (O16) Feeling hopeless and considering the disease as untreatable. [U]  (H14) children became anxious and stressed which affected their school life. Moreover, they had to struggle much on their own. [U] | **Distress and helplessness**  Participants overwhelmingly reported feelings of distress, “*felt unbearable and were filled with dismay*” (J9) and hopelessness for their futures, as they struggled to see their loved one’s struggle with utmost discomfort from an overwhelming disease. | **Synthesised finding 3 - Consequences of caregiver burden**:  There were consequences felt because of their caregiver burden. Taking on the supportive caregiving role meant confronting that patient prognosis can be poor. Caregivers battle with feelings of helplessness, distress, anger and guilt and a strong fear of losing the patient.  Conversely, there is potential for the caregiver to experience growth and feelings of hope |
| (D2) Fear of disease disclosure [U]  (D3) Fear of loss of social status. [C]  (F13) It became a way to avoid guilt or reproach from the survivors or other family members if the survivors got worse. [U]  (I25) Caregiver guilt was experienced [U]  (I39) For some, anger surfaced [U]  (J2) patient’s signs and symptoms of HCC, disease exacerbated and treatment delayed. Therefore, participants felt guilty and blamed themselves. [U]  (N6) Two posters personified pancreatic cancer [U]  (R7) A few family caregivers mentioned the stigma of an HCC diagnosis and substance abuse. [U]  (R8) Felt compelled to inform friends and family that the patient’s diagnosis was not from alcohol abuse. [U] | **Anger and guilt**  Some caregivers reported shame/guilt in being associated with the disease as their culture views a diagnosis of cancer with stigma.  Participants also reported feeling anger*, ‘‘have been dealt a bad hand,’’* (I39) towards the disruption of an UGIC diagnosis. |  |
| (B25) concern about their own future health and that of other family members. [U]  (B3) Fear of the disease and what the future might bring coupled with an overall sadness [U]  (C11) Struggled with thoughts and possible worries about the future. [U]  (C14) struggled to keep a sense of perspective and felt obliged to be the anchor in the uncertainty regarding the future and possible treatment. [U]  (C17) could not handle the uncertainty on a long-term basis but needed to take it day by day. [U]  (C9) suppressed their own anxiety and doubt about the treatment and future [U]  (D10) Fear of mental reactions. [U]  (D11) Fear of physical reactions. [U]  (D12) Fear of emotional changes [U]  (D13) The fear of appearance changes and the effect of different drugs and treatments on the appearance of patient caused distress in the patient family. [U]  (D14) fear of weight loss [C]  (D4) fear of difficulty of treatment - Some participants feared from long-term treatment of the disease and therapeutic follow-ups. [U]  (D7) following cancer diagnosis was mental changes that caused mental distress in participants. [U]  (D8) Fear of cancer.  Involved experiencing different stresses in relation to this disease [esophageal cancer] [U]  (E5) Some family caregivers were still actively involved in the caregiver role while others were trying to get back to a sense of normality. [C]  (F39) Were afraid of their partner’s death and subsequent life without a partner [U]  (F6) GI problems further made the couples frightened, because they regarded them as indicators of recurrence of cancer [U]  (H3) in connection with patients receiving their diagnosis, they experienced a delay in receiving a correct diagnosis [U]  (H16) The family members experienced an everyday symptomatic uncertainty and looked for signs for deterioration. [U]  (H17) A prognostic uncertainty is a medical reality in patients with oesophageal cancer [C]  (H18) The uncertainty of death and dying pervaded the family members’ thoughts and plans for the future. [U]  (H19) The family members expressed a genetic threat and concerns about the connection between genetics and cancer. [U]  (H20) the family members had different strategies for managing uncertainty. [C]  (H41) The family members were also afraid of what they might find. [U]  (I28) for many caregivers, the ‘‘fear of being alone’’ was a driving force for ‘‘holding on.’’ [C]  (K1) Throughout illness and treatment, relatives lived with anxiety and fear of losing the patient. [C]  (K2) They had concerns about the future and were more affected emotionally than the patient. [U]  (K8) Some relatives expressed their sorrow and possible loss of togetherness [C]  (K9) Relatives were always alert and trying to adjust when something unexpected happened. [C]  (L1) Caregivers feared losing their loved ones [U]  (L11) Although they were excited that the patient was doing well, they were also sceptical of the situation, even when presented with good news from the medical team. [U]  (O2) The role of hereditary factors in incidence of disease [U]  (R13) As HCC progressed and patients’ symptoms worsened, family caregivers were uncertain about whether symptoms they could identify were related to the cancer. [U] (R18) When patients’ condition worsened and TACEs were no longer effective, family caregivers contemplated the potential impact of chemotherapy and whether patients would be able to survive. [C] | **Fear of cancer progression and recurrence**  Caregivers reported living in constant fear of the patient’s health declining beyond repair, and referenced specific physiological signs fears (D14), alongside general fears due to the unpredictability of cancer (H14). |  |
| (A3) Carer’s feelings of isolation [U]  (B15) Many participants, however willing to discuss emotions with the interviewer, confessed to having hidden their feelings during the time of crisis [C]  (D1) Fear of isolation and loneliness. [C]  (E11) Family caregivers of non-English-speaking patients experienced a greater sense of isolation as they perceived that language difficulties limited access to information and support. [U]  (E13) focus on patient care, often to the exclusion of self-care, was a major contributing factor to family caregivers’ experience of stress. [C]  (E14) study interview was the first time they had reflected on their own feelings [U]  (I16) Caregivers’ self-care was not a priority. [U]  (I61) all participants welcomed the opportunity to share their true feelings, one going so far as to say that participating in the study ‘‘was a blessing.’’ [U]  (K10) Sometimes patients with EC fought single-handedly with their fears [C]  (K11) When patients withdraw from the family, relatives were precluded from sharing their fear leading to feelings of isolation and loneliness. [C]  (K4) [Relatives] were often isolated with their anxiety, always in consideration for the patient. [U]  (L7) Although caregivers recognized the need for self-care, they tended to “put ourselves on the back-burner” (Helen) [U]  (M14) The spouses stated that they did not want to be left alone in life and they wondered if they would be capable of organizing all the practical things. [U]  (M34) The informants tried to carry their burden all alone. [U]  (S17) Some re-sorted to alcohol to alleviate the stress, while others withdrew from socializing. [U] | **Isolation and loneliness**  Caregivers commonly reported feeling isolated and lonely as they felt they could not share their stresses with others, “The worries were terrible. I went for walks for hours, sobbing uncontrollably” (K10) |  |
| (B12) Make more time for the ill relative. [U]  (B14) The increased time spent benefited the relationships for those with surviving relatives. [U]  (B26) many individuals resolved to positively change their behaviour [U]  (B28) newfound awareness of their own mortality and a new appreciation for the value of their own lives [C]  (B29) less worry with the passage of time [with surviving relative] [U]  (B32) Individuals saw pancreatic cancer as having an impact on their lives for many years to come. [C]  (H13) Family members in this study emphasized the importance of including the whole family in the care given, even the children, whatever their level of knowledge or ability to understand are, because the children were aware that a tremendous change had occurred in the family. [C]  (H15) Crucial for the family members was that their children should participate in information giving. Participation could facilitate the children’s preparedness. [C]  (I37) Caregivers’ identity and meaning and purpose in life were intimately tied to the relationship with the patient [C]  (I46) the idea of coping was not an active process but something ‘‘you just have to do what other choice do you have.’’ [U]  (I49) participants discovered their own strength, experienced the support of others, appreciated time together, were strengthened by the patients themselves, and had a different perspective on life. [C]  (I50) Participants captured a central idea of ‘‘just appreciating being together’’ for whatever time was left. [U]  (I53) Personal growth was expressed as caregivers experienced new perspectives about life. [C]  (I58) a major transition was coming to a point of acceptance that death was near and patients were ready themselves to ‘‘let go.’’ [U]  (K13) Relatives acknowledged the necessity of considerations about possible death. [U]  (K5) Relatives were confronted not only with the mortality of the patient but also with their mortality. [U]  (K7) Confronting and talking about the worst-case scenario helped relatives deal with EC and treatment. [U]  (M18) their marital relationship had improved or that they finally got peace in their life. [U]  (M19) One just has to accept it or that the illness was part of the suffering and misfortune of normal life that one has to reckon with. [U]  (N13) the acceptance of pancreatic cancer or impending death as being the will of God. [C]  (O7) Lack of nutritional self-care [drinks tea hot, but now after his disease he tries to break this habit] [C] | **Personal Growth**  Some couples reported their relationships grew stronger and were *“even closer”* (M18) and participants observed improvements within themselves. *“participants discovered their own strength”* (I49). |  |
